# Enhanced Salt Removal by Unipolar Ion Conduction in Ion Concentration Polarization Desalination

**DOI:** 10.1038/srep25349

**Published:** 2016-05-09

**Authors:** Rhokyun Kwak, Van Sang Pham, Bumjoo Kim, Lan Chen, Jongyoon Han

**Affiliations:** 1 Department of Mechanical Engineering, Massachusetts Institute of Technology, 77 Massachusetts Avenue, Cambridge, MA 02139, USA; 2 Department of Electrical Engineering and Computer Science, Massachusetts Institute of Technology, 77 Massachusetts Avenue, Cambridge, MA 02139, USA; 3 Singapore-MIT Alliance for Research and Technology (SMART) Centre, Singapore; 4 Department of Biological Engineering, Massachusetts Institute of Technology, 77 Massachusetts Avenue, Cambridge, MA 02139, USA

## Abstract

Chloride ion, the majority salt in nature, is ∼52% faster than sodium ion (*D*_Na+_ = 1.33, *D*_Cl−_ = 2.03[10^−9^m^2^s^−1^]). Yet, current electrochemical desalination technologies (*e.g.* electrodialysis) rely on bipolar ion conduction, removing one pair of the cation and the anion simultaneously. Here, we demonstrate that novel ion concentration polarization desalination can enhance salt removal under a given current by implementing unipolar ion conduction: conducting only cations (or anions) with the unipolar ion exchange membrane stack. Combining theoretical analysis, experiment, and numerical modeling, we elucidate that this enhanced salt removal can shift current utilization (ratio between desalted ions and ions conducted through electrodes) and corresponding energy efficiency by the factor ∼(*D*_−_ − *D*_+_)/(*D*_−_ + *D*_+_). Specifically for desalting NaCl, this enhancement of unipolar cation conduction saves power consumption by ∼50% in overlimiting regime, compared with conventional electrodialysis. Recognizing and utilizing differences between unipolar and bipolar ion conductions have significant implications not only on electromembrane desalination, but also energy harvesting applications (*e.g.* reverse electrodialysis).

In conventional electrochemical desalination systems such as electrodialysis (ED)(Fig. 1a)^1^,^2^,^3^ and capacitive deionization (CDI)^4^,^5^, both cations and anions are removed from opposite sides either by ion exchange membranes (IEMs) or ion selective electrodes. By the requirements of electroneutrality and current conservation, such systems need anion and cation fluxes through the permselective walls to be equal (*J*^+^_*CEM*_ = *J*^*−*^_*AEM*_, Fig. 1a); and the amount of desalted ions is equal to that of ions passed through the membranes. Then, two efficiency parameters of desalination−current utilization (CU) and energy per ion removal (EPIR)−are fixed for ideal systems: CU = 1 and EPIR/V^*^ = 1 (SI Fig. 1), where


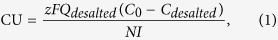



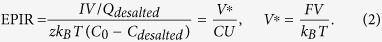


*z*, *F*, *k*_B_*T* indicate ion valence, Faraday’s constant (=9.65 × 10^4^ C·mol^−1^), and thermal energy (=2.479 kJ/mol, *k*_B_ and *T* are Boltzmann constant and temperature) respectively. *V* is voltage, V^*^ is nondimensionalized voltage, *I* is current, *N* is the number of membrane pairs, *C*_0_ is initial ion concentration, *C*_desalted_ is ion concentration of desalted flow, and *Q*_desalted_ is the total desalted flow rate. EPIR is representing how efficiently energy is consumed to reject ions by combining the concept of energy consumption (=*IV/Q*_desalted_) and salt removal ratio (=(*C*_0_ − *C*_desalted_)/*C*_0_). (See SI Section 1.5 for a discussion on the definition of CU). Inevitable loss mechanisms in real systems (*e.g.* imperfect permselectivity of IEMs, current leakage, undesirable chemical reactions such as water splitting) drops CU below 1^1^, and increases EPIR/V^*^ above 1. According to equation (2), at the given ratio by determined CU, energy efficiency (represented by EPIR) improves as V^*^ is decreased, at the cost of slower ion removal at low driving electric field; slower ion removal requires lower operating flow rate or larger system. This trade-off between production capacity and energy efficiency is indeed universal, and can be found in other desalination methods (*e.g.* applied pressure (flow rate) vs. energy in reverse osmosis)^6^.

Escaping from this trade-off issue, in this paper, we show that one can achieve higher salt removal ratio in ion concentration polarization (ICP) desalination than in ED by relying on unipolar ion conduction with only cation exchange membranes (CEMs) (Fig. 1b). Theoretical analysis, experiment, and numerical model accurately capture that salt removal and CU can be enhanced by the factor ∼(*D*_−_ − *D*_+_)/(*D*_−_ + *D*_+_). Specifically for desalting NaCl, ∼20% CU enhancement of unipolar cation conduction saves ∼50% power consumption compared with conventional ED.

## Theory

**Figure 1:**
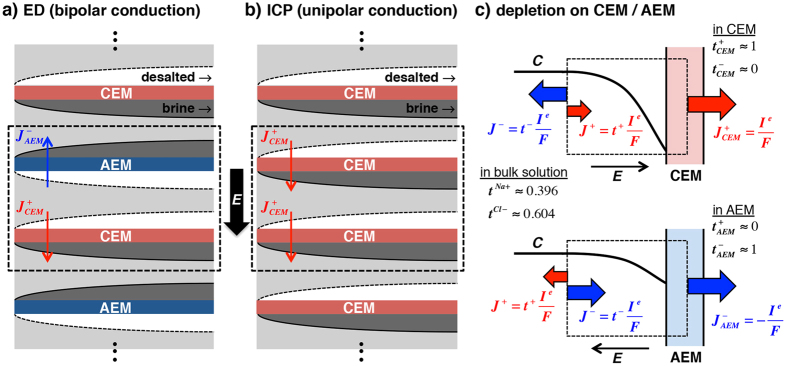
Standard bipolar ED vs unipolar ICP desalination. (**a**) Standard bipolar ED and (**b**) unipolar ICP platforms have desalted flows with low ion concentration at the anodic side of CEMs and at the cathodic side of AEMs (white regions); and *vice versa* for brine flows (dark gray regions). ICP platform is also built with AEMs but the location of desalted/brine flows would be reversed. Blue and red arrows indicate the ion flux through the membranes. The black dotted boxes are the one membrane pair (*N* = 1) for ED and ICP systems, which are repeated; both are functionally matched (membrane number, flow rate, water recovery, *etc.*). (**c,d**) Schematic diagrams demonstrating current (charge) conservation near the CEM (**c**) and the AEM (**d**). The dotted box represents the control volume, at which boundaries current conservation is considered. On the right boundaries (within the membrane), ion flux (*J*^+^_CEM_ or *J*^−^_AEM_) is predominantly carried by one kind of ions; here, we assume the perfect permselectivity of the membranes (*i.e.* transference number at CEM: *t*^+^_CEM_ ∼ 1, *t*^−^_CEM_ ∼ 0 and transference number at AEM: *t*^+^_AEM_ ∼ 0, *t*^−^_AEM_ ∼ 1^36^) (with the net system current *I*^e^).

When ions pass through IEMs selectively, dynamic changes of ion concentration occur near the membrane to maintain electroneutrality against cation or anion-biased transport. This phenomenon is called ICP (also known as ion depletion and ion enrichment)^7^,^8^,^9^. As per directions of the membranes’ biased transport, ion depletion occurs on the anodic (cathodic) side of the CEM (anion exchange membrane (AEM)) (Fig. 1). In this scenario, the strength of ion depletion on the CEM and the AEM are different, because cation and anion diffusivities are different^10^,^11^,^12^; accordingly, these CEMs and AEMs are not equally contribute to desalination. We can glance this unsymmetrical phenomenon in previous ED with stronger depletion on CEMs than that on AEMs^12^,^13^. As one can see in Fig. 1c, when a given current is passed through the CEM (*I*^*e*^), within the CEM, this is entirely carried by cations moving to the other side of the CEM, creating a depletion region (black curved line, Fig. 1c). In order to make the control volume (black dotted line, Fig. 1c) electroneutral, the same amount of current (*I*_*e*_) should be conducted through the left side of the control volume, where both anions and cations are carriers (in bulk solution). In the membrane, this current consists of counter-ion flux (*J*^+^_CEM_ = *I*^e^/*F*), yet in the bulk solution only a part of that current (*t*^+^*I*^e^/*F*) are carried by the counter-ion conduction, and the other part (*t*^−^*I*^e^/*F*) is carried by the co-ions. This unmatched ion carriers results in the depletion of ions from the control volume by the amount of *t*^−^*I*^e^/*F*. *t*^±^ is the transference number for dilute binary electrolytes in bulk solution, defined with cation/anion diffusivities (*D*_±_) as *t*^±^ = *D*_±_/(*D*_+_ + *D*_−_). We assume that electromigration is a dominant transport mechanism in bulk solution; the transference number is only the function of salt ion diffusivities. Therefore, the depletion of ions near the membrane, whether that is the CEM or AEM, is determined by the amount of the co-ions (anion for CEM an cation for AEM) that are moving away from the membrane (without such movement, there cannot be any depletion and desalination). If one have NaCl as majority salts (with significantly higher diffusivity of Cl^−^ compared with Na^+^, *t*^Na+^∼0.396 < *t*^Cl−^ ∼ 0.604)^14^, on the CEM (Fig. 1c), electroneutrality can be met only by removing more Cl^−^ away from the depletion zone, and less ‘back-filling’ of Na^+^ ions into the depletion zone. As a result, the amount of depleted ions on the CEM is *J*^Cl−^ = *t*^*Cl*−^*I*^e^/*F* = 0.604 *I*^e^/*F*. In the case of the AEM, in Fig. 1d, the co-ion (Na^+^) diffusivity is less than that of conducting ions (Cl^−^), generating a weaker depletion zone than that on the CEM with the less amount of depleted ions, *J*^Na+^ = *t*^*Na*+^*I*^e^/*F* = 0.396 *I*^e^/*F*. If one have KCl salt (almost equal mobility of cations and anions, *t*^K+^ ∼0.49 and *t*^Cl−^ ∼ 0.51)^14^, in the bulk region, approximately half the current is carried by K^+^ (therefore making up lost cations in the depletion region), another half carried by Cl^−^. Then, the strength of depletion zone on the CEM and the AEM are similar. The analytical solution and microscopic observation by simulation offer a solid support to this explanation (SI Section 1,2).

Revisiting ED system with this microscopic view, one ED membrane pair (one CEM and one AEM) can remove the amount of ions *J*^+^ + *J*^*−*^, where *J*^+^ = *t*^+^*I*^e^/*F* by the depletion on the AEM and *J*^*−*^ = *t*^−^*I*^e^/*F* by the depletion on the CEM (black dotted box, Fig. 1a). In ED system, then the depleted ions *J*^+^ + *J*^*−*^ is always *I*^e^/*F* (=*J*^+^_CEM_ = *J*^*−*^_AEM_) because *t*^+^ + *t*^*−*^ is always 1, regardless of salt ions and their mobilities. Finally, we reach the conventional result that the CU of ED process (=desalted ions/conducted ions = (*J*^+^ + *J*^*−*^)/*J*^+^_CEM_) is always one. In ICP desalination, one utilizes ICP zones from either only CEMs (termed as ICP − CEMs) or only AEMs (termed as ICP − AEMs) (Fig. 1b), forcing the system to rely on unipolar ion conduction. By this, we can choose ‘stronger’ depletion zones (*e.g.* depletions near the CEMs, for NaCl removal). In the ICP − CEMs process, the amount of removed ions by one membrane pair (now with two CEMs, instead of one CEM and one AEM) is 2*J*^*−*^ (instead of *J*^+^ + *J*^*−*^), where one *J*^*−*^ = *t*^−^*I*^e^/*F* by the one depletion on the single CEM (black dotted box, Fig. 1b). Then, with the removed ions 2*J*^*−*^ and the transferred ions *I*^e^/*F* (=*J*^+^_CEM_ = *J*^*−*^_AEM_), the CU of ICP − CEMs process becomes 2*J*^*−*^/*J*^+^_CEM_ = 2*t*^*−*^. Finally, we obtain the theoretical CU (and EPIR/V^*^) for unipolar ICP desalination from this control volume analysis, which is not one. For general binary electrolytes,


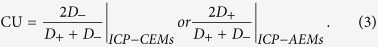



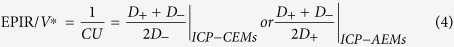


It is noted that this control volume analysis is acceptable for any current regimes with thin, thick, or even overlapped ICP zones in ED, as long as the desalted and brine flows are separated (we present the ICP system with hydrodynamically separated desalted/brine flows in Discussion). This equation (3) shows that ICP desalination process has the different limit of CU, which can be above or below 1 depending on salt ions in water (*e.g.* CU = 1.208 for NaCl with ICP − CEMs, where *D*_Na+_ = 1.33, *D*_Cl−_ = 2.03 [10^−9^ m^2^ s^−1^]^14^). As CU is enhanced in ICP desalination, from equation (4), EPIR/V^*^ can be below 1 (*e.g.* EPIR/V^*^ = 0.827 for NaCl with ICP − CEMs) (see SI for detail derivations of CU and EPIR/V^*^). In contrast, the best EPIR/V^*^ values achieved even in the limit of incremental salt removal ratio (with minimized entropy loss) in membrane CDI is still above 1 (EPIR ∼ 50*k*_B_*T*, *V* = 1.2V, EPIR/V^*^ ∼ 1.07)^5^. ICP desalination can provide competitive advantages in terms of reducing EPIR and power consumption.

**Figure 2:**
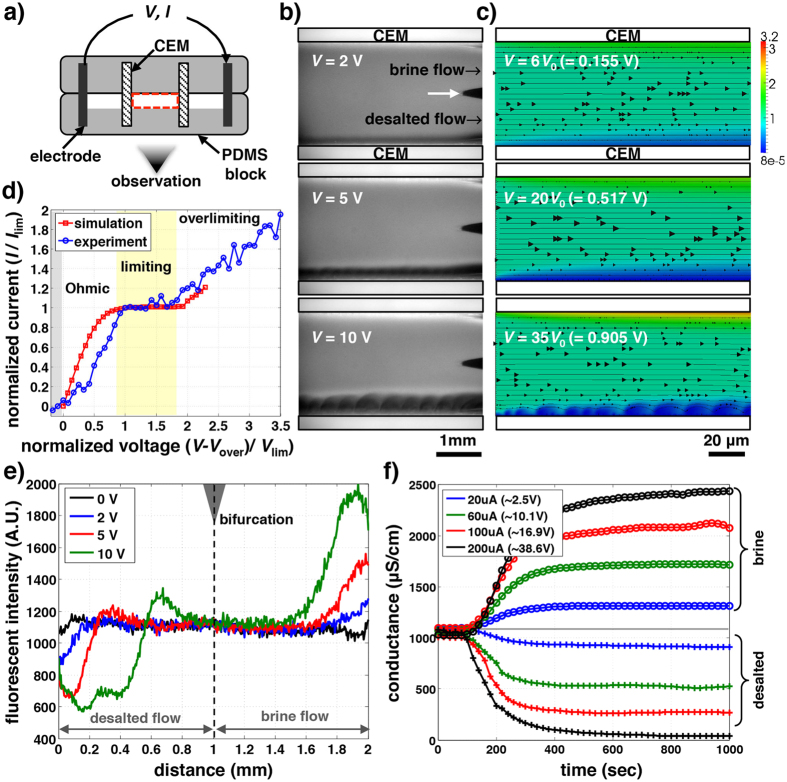
ICP phenomenon in unipolar ICP desalination. (**a**) Schematic of micro-ICP desalination platform. Two CEMs and two electrodes are inserted in the transparent PDMS blocks to observe *in situ* dynamics. Three channels are built: one channel between the two CEMs, which are bifurcated as the desalted and brine flows at the end of the channel, and two electrode channels where Faradic reactions happens. (**b**) Experiment and (**c**), numerical simulation demonstrate ICP desalination. In simulation (**c**), colar bar and black arrows indicate ion concentration and streamlines. The visualized experiments are also available for various electrolytes and the other systems (ICP − AEMs and ED)(SI Figs 7 and 9). The detailed experiment and simulation methods are described in Methods and Kwak *et al.*^13^,^15^. (**d**) Current-voltage curves of ICP − CEMs in experiment and simulation. The curves are normalized with the limiting voltage *V*_lim_ and current *I*_lim_ (experiment: *V*_lim_ = 3 V, *I*_lim_ = 3.63 × 10^−5^, simulation: *V*_lim_ = 14 *V*_0_, *I*_lim_ = 0.272*I*_0_, where *V*_0_ = 25.85 mV, *I*_0_ = 330 μA). The current-voltage response is measured experimentally by ramping up the voltage by discrete voltage jumps of 0.2 V in every 30 seconds. (**e**) fluorescent profiles right before the bifurcation and (**f**) conductance measurements (operated by constant current). The conductance of the desalted flow decreases up to 97% at 200 μA (38.6 V). The voltage response is measured during 300 sec and averaged. The dye accumulation on this strong depletion zone (10V (**b**,**e**)) is due to preconcentation effect by its electrophoresis (no salt accumulation)^9^.

In the above analysis, one membrane pair (*N* = 1) of ICP − CEMs is selected to be the *functional equivalent* of the one membrane pair of ED (black dotted boxes in Fig. 1a,b). Therefore, ED and ICP − CEMs with the same membrane pairs have the same amount of membranes used (which is the main cost driver for electrochemical desalination systems), the same input feed water flow and the output desalted/brine flow rates, and the same operating current (*I*^e^) with the same recovery ratio of 50%, and the same inter-membrane distance resulting in the same size of the system, and the same flow velocity profile between the membranes. The only difference of ICP system from ED is that the two desalted/brine flows, induced from the adjacent two membranes, do not occur within the same channel. Instead, ion depletion and ion enrichment zones are generated within a single channel, so we separately collect the desalted flow and the brine flow by bifurcating the channels at the end (Fig. 2a,b).

## Results

Firstly, with the simple ICP desalination unit, we confirm that unipolar ion conduction can desalinate salts using recently developed micro ED platform^13^,^15^ (Fig. 2a,b) as well as multiscale numerical simulation^13^,^16^ (Fig. 2c). Micro ED platform can visualize *in situ* fluid flow and ion concentration while controlling or measuring all relevant parameters (*e.g.* voltage, current, conductivity) simultaneously. In experiment, ICP desalination is observed between two juxtaposed CEMs. The flow between them is bifurcated at the end as one desalted flow and one brine flow (white arrow, Fig. 2b). We also demonstrate the numerical simulation to reproduce experiments (Fig. 2c). In Fig. 2d, ICP desalination shows a similar current-voltage response and flow/concentration profiles as conventional ED, which can be categorized as Ohmic (experiment: 0.6–3 V, simulation: 0–14 *V*_0_, *V*_0_ = *k*_B_*T*/*e* = 25.85 mV), limiting (experiment: 3–4.4 V,simulation: 14–26 *V*_0_) and overlimiting regimes (experiment:>4.4 V, simulation:>26 *V*_0_)^17^,^18^. Overpotential by Faradaic reaction and/or contact resistance exists in experiments with the current below zero (0–0.6 V). As described by the conventional model of ICP^7^, linear concentration gradient near the membranes is observed in Ohmic regime (experiment: 2 V in Fig. 2b,e/simulation: 6 *V*_0_ in Fig. 2c). When the ions at the membrane surface nearly vanish, the concentration gradient and corresponding current are saturated, resulting in a plateau on the I-V curve (also known as limiting current)(experiment: 5 V in Fig. 2b,e/simulation: 20 *V*_0_ in Fig. 2c). In overlimiting regime, however, nonlinear driving force from extended space charge layer generates sheared electroconvection, and results in a flat depletion zone with a extremely low ion concentration (experiment: 10 V in Fig. 2b,e/simulation: 35 *V*_0_ in Fig. 2c)^13^,^15^,^19^,^20^. To quantify the desalting performance, we measure the conductivities of bifurcated desalted and brine flows by connecting a flow-through conductivity probe at the outlets (Fig. 2f). After the processed water fills the whole volume of the probe (∼17 μL), the conductance of the desalted (brine) flows maintains the lower (higher) value stably, representing steady-state ICP desalination achieved. It is also noted that; i) the conductance curves are symmetric, indicating ions removed from the desalted flow relocate to the brine flow (ion charge in the channel is conserved, even with CU over 1); ii) salt removal ratio can be achieved up to ∼97% with the flat depletion zone at overlimiting regime (200 μA, Fig. 2f). If concentration profile is linear as expected by conventional ICP theory^7^, the highest salt removal would be 50%.

**Figure 3:**
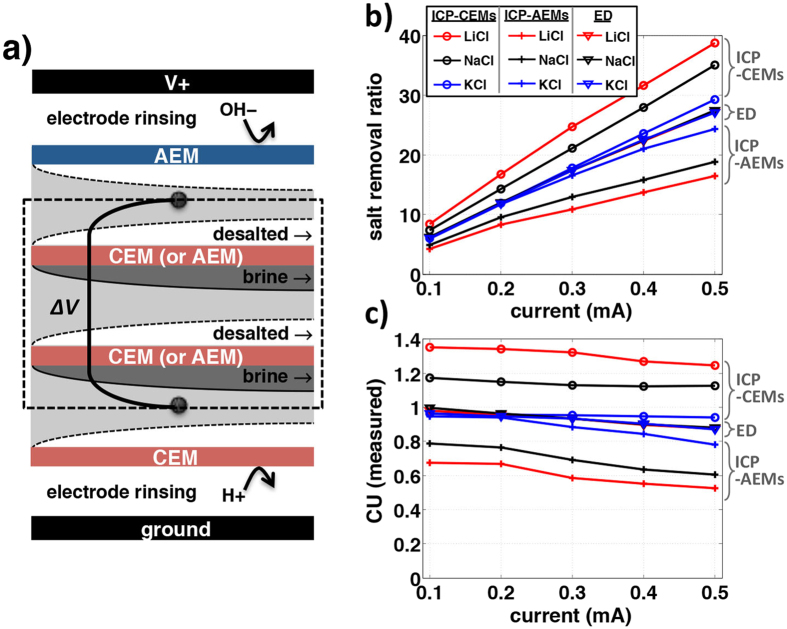
Salt removal ratio and current utilization (CU) in ED and ICP systems. (**a**) Schematic setup of comparative experiment (ICP − CEMs). On the one membrane pair of ED or ICP (black dotted box), AEM and CEM are added to cover possible byproducts on electrodes, especially protons and hydroxide ions. By replacing one CEM to the AEM, we obtain the ED system; while ICP − AEMICP − AEMs is available by replacing both two CEMs (SI Fig. 10). (**b,c**) At the various applied current (0.1–0.5 mA), salt removal ratio (%) (**b**) and CU (**c**) of ED and two ICP are measured according to three different salts. We use 10 mM LiCl, NaCl, and KCl, where each ion diffusivity is *D*_Li+_ = 1.03 <*D*_Na+_ = 1.33 <*D*_K+_ = 1.957 <*D*_Cl−_ = 2.03 [10^−9^ m^2^s^−1^]. The flow rates between the IEMs or on the electrodes (rinsing) are 100 or 500 μL/min, respectively. Conductance of desalted flow is read out after it is saturated (Fig. 2f). Current-voltage curves (SI Fig. 12a–c) are available in SI.

**Figure 4:**
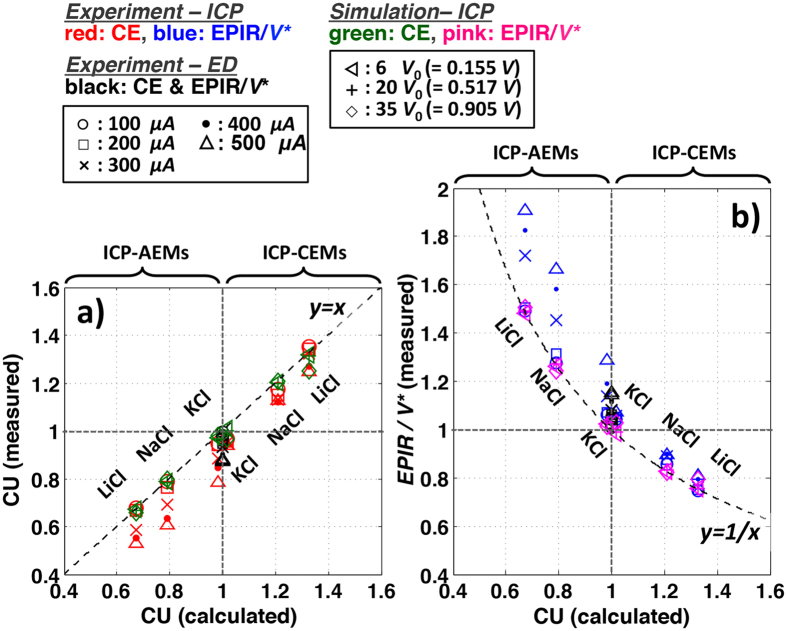
CU and EPIR shifts by salts’ inherent asymmetry. (**a,b**) Measured CU (**a**) and EPIR/V^*^ (**b**) of ICP and ED systems are plotted versus the theoretical values given by equation (3, 4). The measured CU and EPIR/V^*^ are obtained by the experimental dataset in Fig. 3 and the numerical simulation (SI Fig. 7). Constant current in experiment (Ohmic and limiting: 0.1–0.2 mA, overlimiting: 0.2–0.5 mA) and constant voltage in simulation (Ohmic: 6*V*_0_, limiting: 20*V*_0_, overlimiting: 35*V*_0_, *V*_0_ = 25.85 mV) are applied. For ICP desalination systems, equations of best fitting lines for each experimental date sets are y = 1.077x + 0.147(*R*^2^ = 0.94, red (**a**)) and y = 1.07(x − 0.146)^−1^ (*R*^2^ = 0.94, blue (**b**)). For simulation data, the best fitting lines are y = 0.966x + 0.0195(*R*^2^ = 0.99, green (**a**)) and y = 1.006x^−1^ (*R*^2^ = 0.99, pink (**b**)). CU and EPIR/V^*^ of ED system is on the (1,1) (black (**a**,**b**)).

Secondly, to confirm CU and EPIR/V^*^ shifts in unipolar ICP desalination, we compare two ICP desalination modalities (CEMs or AEMs only) with standard ED process (Figs 3 and 4). Salt removal ratio, CU, and EPIR/V^*^ of ICP/ED systems were experimentally measured and also calculated (by numerical simulation) with monovalent binary electrolytes (NaCl, KCl, and LiCl) in various current regimes (experiment: 0.1∼0.5 mA, simulation: 6–35 *V*_0_, where *V*_0_ = 25.85 mV). Here, the AEM (CEM) is added on the anodic (cathodic) side to block protons and hydroxide ions produced by Faradaic reactions on electrodes, which can affect the conductance in the main channel significantly due to their high diffusivities (*D*_H+_ = 7.99, *D*_OH−_ = 5.26[10^−9^m^2^/s]^14^) (Fig. 3a). The two electrodes apply a constant current, and the resulting voltage difference is read out at the two probing electrodes (black dotes, Fig. 3a). Such experimental set-up has been previously used to characterize ED membrane performances without being affected by any overpotentials and other irregularities at the electrodes^21^,^22^,^23^,^24^. As expected by equations (1) and (3), when *D*_−_ > *D*_+_ (NaCl and LiCl), salt removal ratio and the CU of ICP − CEMs shift up from the values of ED, while they shift down in ICP − AEMs (Fig. 3b,c). When *D*_−_ ∼ *D*_+_ (KCl), the shifts are much smaller and negligible; split over at >0.2 mA probably because of non-ideal effects (*e.g.* water splitting, fouling)(we will discuss this effect at the next section). All CU and EPIR/V^*^ values are then collapsed onto theoretical lines (dotted lines in Fig. 4a,b, error: <6% in experiment, <1% in simulation), validating equation (3,4) from both experiments and simulation models. These lines and data meet at (1,1), which represents the values for conventional ED. There is no CU and EPIR/V^*^ shifts from ED without salts’ asymmetry (*D*_−_ = *D*_+_), as can be expected. The amount of this CU shift (and following EPIR/V^*^ shifts) of ICP desalination is represented by only salts’ inherent asymmetry, (*D*_−_ − *D*_+_)/(*D*_−_ + *D*_+_) (=|CU_ICP_ − 1|).

**Figure 5:**
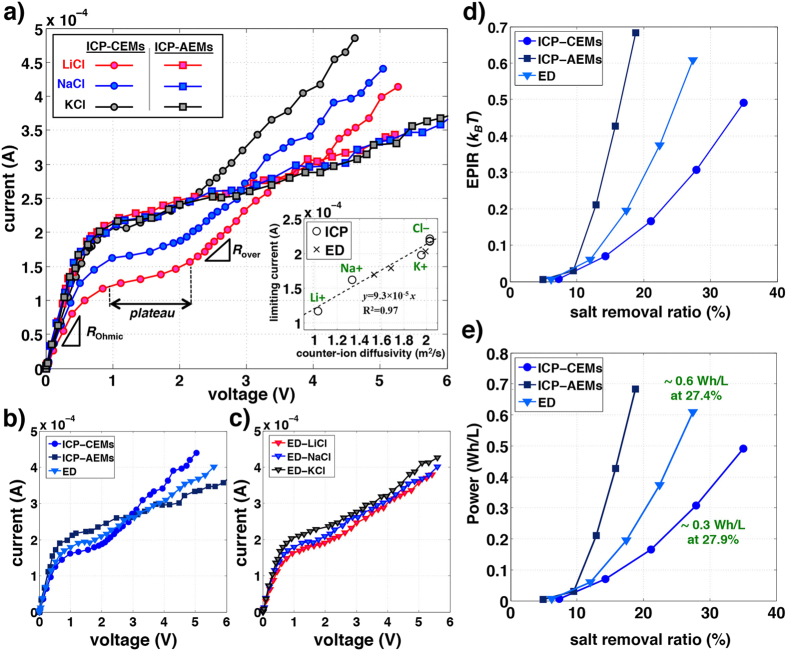
Current-Voltage (I-V) response and energy analysis. (**a–c**) Current-voltage (I-V) curves of ICP − CEMs (circles), ICP − AEMs (squares), and ED (triangles) with various salts: 10 mM KCl (black lines), NaCl (blue lines), and LiCl (red lines). The current responses are measured by ramping the current up with 0.5 V steps from 0 to 10 V with 40 sec delay. In these curves, we define the limiting current (inset graph (**a**)), Ohmic resistance *R*_Ohmic_ (SI Fig.10a), the plateau length of limiting regime (SI Fig.10b), and overlimiting resistance *R*_over_ (SI Fig.10c). Limiting currents is governed by counter-ions (cations in ICP − CEMs and, anions in ICP − AEMs); the counter-ion diffusivity of ED is assumed as the mean diffusivity of cation and anion, (*D*_+_ + *D*_−_)/2. (**d,e**) For all three systems (ICP − CEMs: circles, ICP − AEMs: squares, ED: triangles) in 10 mM NaCl solution, EPIR (**d**) and power consumption (**e**) according to salt removal ratio are shown. The dataset is used in Figs 3 and 4 (and SI Fig. 12). As can be seen, the power consumption of ICP − CEMs (0.3067 Wh/L at 27.9% of the salt removal ratio) is significantly reduced than that of ED (0.6083 Wh/L at 27.4% of the salt removal ratio).

Next, we investigate that CU and EPIR/V^*^ shifts of ICP desalination can provide any reduction in EPIR and power consumption. To this end, current-voltage (IV) responses are measured for unipolar ICP desalination (either with CEMs or AEMs) as well as bipolar ED system experimentally (Fig. 5a–c). The IV curves of ED process are always between the curves of ICP − CEMs and ICP − AEMs (Fig. 5b). This indicates ICP zones on CEMs or AEMs are indeed independent in this experiment. In Ohmic and limiting regimes (0–2 V, Fig. 5a–c), limiting current and 1/*R*_Ohmic_ are known to be linearly proportional to the diffusivity of conducting ions (counter-ions)^7^,^11^,^14^, which is reproduced in our experiment (inset image in Fig. 5a and SI Fig. 10) and the analytical solution (equation SI 2.26). The current through the CEM (*I*_+_) or the AEM (*I*_−_) is *I*_±_ ∼ V^*^(*D*_±_/*D*_eff_), where *D*_eff_ = 2*D*_+_ *D*_−_/(*D*_+_ + *D*_−_). The limiting current can be read at the location where the current-voltage curve is departing from Ohmic characteristics^2^,^25^ (∼1 V, Fig. 5a–c). As a result, with slower cation diffusivities (*D*_Cl−_ > *D*_K+_ > *D*_Na+_ > *D*_Li+_), ICP − CEMs has higher *R*_Ohmic_ than ICP − AEMs and ED (and higher V^*^ requirement), which counteracts the enhanced salt removal ratio and CU at a given current (ICP − CEMs: V^*^ ∼ *R*_Ohmic_∼1/*D*_+_, ICP − AEMs: V^*^ ∼ *R*_Ohmic_ ∼ 1/*D*_−_). Therefore, regardless of electrolytes, experimental EPIR and power consumption are similar to all ICP and ED systems in Ohmic regime (<0.2 mA in SI Fig. 11, <10% salt removal ratio in Fig. 5d,e), as shown in a simple theoretical calculation:


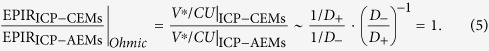


However, in overlimiting regime, the resistance of ICP − CEMs becomes significantly smaller than that of ICP−AEMs and ED, because the slower movement of counter-ions facilitates electroconvection, which is largely responsible for overlimiting conductance (Fig. 5a,b and SI Fig. 11b,c). According to Choi *et al.*^26^ and Rubinstein *et al.*^17^, electroconvective Peclet number (Pe_EC_ ∼ 1/*D*_counter-ion_) indicates both the easiness of vortex initiation, and its strength; the plateau length and *R*_over_/*R*_Ohmic_ are proportional to 1/Pe_EC_. The current-voltage curves of three systems are then crossed at ∼2–3V (Fig. 5a,b). With similar analogy but with different resistance to equation (5), the EPIR ratio of ICP systems is:


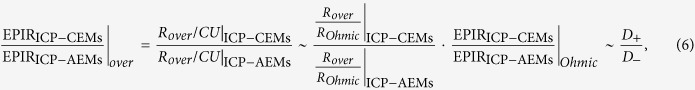


As a result, by means of CU enhancement and lower resistance in overlimiting regime, the EPIR and power consumption (≈EPIR × salt removal ratio) of ICP − CEMs are smaller than that of the other two (*D*_+_/*D*_−_ <1) (>0.2mA in SI Fig. 11, >10% salt removal ratio in Fig. 5d,e). For desalting NaCl, we can expect that ICP − CEMs reduces EPIR and power ∼35% than ICP − AEMs (=1− *D*_Na+_/*D*_Cl−_) or ∼17.5% than ED (by equation (6)). In experiment, ICP − CEMs shows ∼80% lower values than ICP − AEMs (and ∼50% lower values than ED). This is probably because H^+^/OH^−^ generation is expedited on AEMs caused by water splitting^12^,^27^ and current-induced membrane discharging^28^. In ICP − AEMs and ED, this can decrease salt removal ratio and CU by supplying additional salts (additional downshifts of salt removal ratio (Fig. 3b) and CU (Figs 3c and 4a)). Also, this ion generation can suppress the initiation of electroconvection^28^, resulting in minor resistance change (Fig. 5a) and the additional upshift on *R*_over_/*R*_Ohmic_ (SI Fig. 10c).

## Discussion

The new unipolar ICP desalination can offer advantages in many applications where standard ED is successfully used, including desalination and specific ion regeneration or rejection^2^,^29^, because CU and EPIR/V^*^ improvements by unipolar ICP process are applicable to various multicomponent electrolytes found in these applications (see SI Section 1.3). NaCl is the majority salt in natural water to be desalinated (∼85%)^30^, so CEMs-only ICP desalination (ICP − CEMs) will show ∼20% CU and EPIR/V^*^ enhancements, and 50% energy cost reduction than typical ED (at the same salt removal). Also, for the treatment of other industrial wastewater (*e.g.* produced water from oil/gas industry), ICP process allows us to increase efficiency by choosing the better IEMs (CEMs if Σ*z* _−_ *c* _−_ *D* _−_ >Σ*z* _+_ *c* _+_ *D* _+_ or AEMs if Σ*z* _−_ *c* _−_ *D* _−_ < Σ*z* _+_ *c* _+_ *D*_+_). Given that ED is generally considered a mature technology, this improvement by using salt’s inherent asymmetry and electroconvection has significant practical implications.

We also can apply this unipolar ion conduction reversely for reversible process of ED, *i.e.* reverse ED for energy harvesting from salinity gradient^31^,^32^. In the same analogy (but reversely), with a given NaCl concentration gradient (Δ*C*), AEMs-only reverse ICP system would be harvest ∼25% more energy than conventional reverse ED; current flow is *I* ∼ Δ*C*/CU, so *I* ∼ 1.25Δ*C* with ICP − AEMs (at CU = 0.8), whereas *I* ∼ Δ*C* with ED (at CU = 1). This shift of energy harvesting performance by unipolar ion conduction was qualitatively observed in a nanofluidic reverse ED^33^.

**Figure 6:**
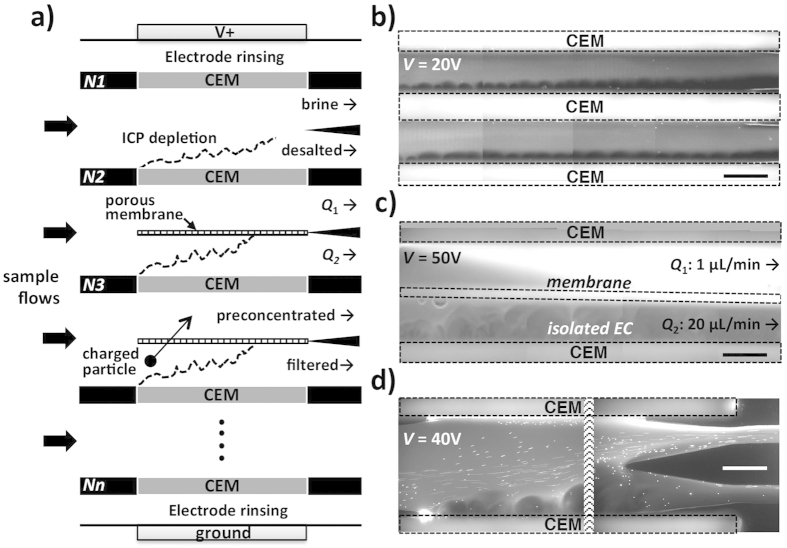
Extended platforms of unipolar ICP system for various applications. (**a**) Multi-stacked (*N1* to *Nn*) multifunctional unipolar ICP desalination platform. Sample flows (thick arrows) are separated into fresh desalted flows and brine flows. Dotted lines (**a**) indicate ion depletion zones to reject ions, which are developed identically on the membranes (2-channel test with 10 μL/min per channel (**b)**). The device in this work has a shallow channel depth of 0.2 mm, which can treat 20 μL/min. For example, if we modify a commercial handheld ED (25 membrane pairs, active membrane area: 64 cm^2^, intermembrane discance: 1 mm, and the total device dimensions 165 × 150 × 190 mm^3^, weight: 3 kg, ED64004, PCCell GmbH, Germany), we can potentially deal with sample water 192 L per 1 hour. 4-channel (2 membrane pairs) test of ICP − CEMs is demonstrated to achieve ∼1 mL/min (SI Fig. 13). High water recovery desalination (*N2* (**a**)) and particle preconcentration (*N3* (**a**)) are also demonstrated. A microporous membrane (meshed box (**a**)) is located between two IEMs to separate desalted/brine channels hydrodynamically. This membrane isolates EC’s instability at overlimiting current regime only in the desalted flow. (**c**) Demonstration of ∼95% water recovery of 10 mM NaCl solution/21-fold preconcentration of Alexa Fluor 488 with ICP − CEMs and 1 μm polycarbonate membrane (Sterlitech Co., Kent, WA). The water recovery/preconcentration factor can be expected as the ratio of desalted and brine flow rates, *Q*_2_/(*Q*_1_ + *Q*_2_) = 0.95 and (*Q*_1_ + *Q*_2_)/*Q*_1_ = 21, respectively. (**d**) Particle removal in ICP − CEMs. Negatively charged Alexa Fluor 488 and 6 μm microparticles (Polyscience, Inc, Warrington, PA) are loaded in 10 mM NaCl solution only on the lower part of the flow. At 40 V, the most negative-charged particles/dyes move upwards. All scale bars (**c,d**) indicates 1 mm.

At the same time, the ICP desalination platform is scalable to larger volume processing in the same manner as the conventional ED. In fact, one can potentially use existing ED platform and modify it into ICP system, by simply removing all CEMs or AEMs yet keeping all the fluid routing (Fig. 6a,b and SI Fig. 13). If one adds a porous membrane between IEMs, which can sustain hydrodynamic pressure difference, water recovery ratio can be controlled by adjusting flow rate ratio between the desalted and brine flows (*e.g.* 95% water recovery, *N*2 in Fig. 6a,c). Therefore, this will make the technology readily implemented to conventional ED systems at various scales. The key difference between ED and ICP systems would be that ICP process requires the brine and desalted streams not to be mixed to sustain CU and salt removal enhancement (so that they can be routed separated in the end or continuously as in Fig. 6a,c); while ED would require the overlapped or mixed boundary layers in the desalted channel to achieve high salt removal. However, as we already addressed, the flat and low ion concentration profile by electroconvection allow us to achieve 97% salt removal without the overlapping or mixing of boundary layers (at 200 μA in Fig. 2f). In addition, this separated desalted/brine flows in one channel enables another unique benefit of ICP process, which is the fact that all the non-salt particulates, often known as Total Suspended Solids elements (*e.g.* cells and colloids), are also comigrate with the brine flows and removed in the same process (*N*3 in Fig. 6a,d and SI Fig. 13)^19^,^34^,^35^.

To apply ICP system in the practical field of generic desalination, we need to consider i) non-ideal effects and capital costs of CEMs and AEMs, and ii) the ICP system’s competitiveness and marketability. First of all, for high salinity applications such as seawater and produced water, its high feed water salt concentration (>500 mM) can be lowered CU by worsening the permselectivity of the membranes (in low salinity solution such as brackish water, the permselectivity is nearly perfect)^23^,^36^,^37^. While this tendency is global, the degree of permselectivity drop on CEMs and AEMs is different according to various factors (external: electrolytes and its concentration, pH and internal: membranes’ material (*i.e.* fixed ionic groups), thickness, conductivity, structure (which determines ion permittivity)). If we have the identical CEMs and AEMs (which are the same permselective properties according to the above factors, except the polarity), the CU shift stays the same even in any cases; because the permselectivity and CU will be rendered in both ED and ICP systems equivalently. If the AEM shows the significant lose of permselectivity in high salinity, ICP − CEMs (operated only with CEMs) will have more enhanced CU than ED over the expected CU shift theoretically (by equation (3)). In addition, when we prefer to use high current regime (*i.e.* overlimiting), the onset of electroconvection and its strength is depend on membranes’ hydrophobicity and heterogeneity^24^,^38^. Another important issue in the practical field is membrane fouling, which is the reason to develop electrodialysis reversal^2^. This fouling problem governs the membranes’ lifetime and corresponding capital cost of the whole system. Typically, Total Suspended Solids (*e.g.* virus, bacteria) in natural water sample are electronegative^39^, so the CEMs is less susceptive to fouling, which is adding to the merit of ICP − CEMs over ED.

Second, electrochemical desalination systems (ED and CDI) have been frequently tested in relatively low salt concentration (1–100 mM); because it is often considered that they are competitive only for brackish water desalination^2^,^3^,^4^. However, the best energy efficiency of reverse osmosis is often achieved in large-scale plant operation with energy recovery systems (all involving significant capital cost), and portable, small-scale RO systems are not as energy efficient as the large-scale plant^3^,^40^. Since ED and ICP technologies are generally requiring lower capital costs, and also scalable (it can be implemented in small and medium scale systems), there is a market opportunity for ED or ICP technologies in small, portable desalination units, even for seawater. In addition, feed water with higher salinity (50 k ppm or more) than seawater is actually precipitously challenging to treat with RO, while electrochemical methods may be the most promising option^3^,^40^. Due to the reasons mentioned above, we believe that it is justifiable to ED/ICP technologies for the treatment of seawater or even brine. There has been a recent surge of study on electrical desalination, spurred by the challenges in treating high concentration brine (such as in produced water from oil/gas industry^41^,^42^). Recently, as a example to show this possibility, Siemens presents the low-energy seawater desalination by combining ED and continuous electrodeionization in 2011^43^.

## Conclusions

In this paper, we firmly identified the scientific principles behind of ICP desalination by modeling and experiments, and studied the key characteristic of unipolar ion conduction compared with standard bipolar ED process. Previously demonstrated ICP desalination systems^35^,^44^,^45^,^46^ had many significant challenges, such as inefficient channel design, exposed electrodes (chlorine species can be generated and mixed with desalted water), and the lack of clear scale-up strategy. In the platform presented here, all the issues described above are resolved while retaining many benefits of ICP desalination processes. Quantifiable energy and efficiency improvements of unipolar ICP desalination over standard ED has been elucidated, with solid scale-up strategy (Fig. 6a and SI Section 7). This enhancement mechanism of unipolar electrical desalination probably can also be applied to other electrochemical systems with IEMs, including CDI^4^,^5^ and reverse electrodialysis^31^,^32^.

## Methods

### Experiment

Experiments were performed using a microscale electrodialysis (ED) platform^13^,^15^. ICP desalination and ED systems were fabricated by slotting anion exchange membranes (AEM), cation exchange membranes (CEM), and two electrodes at the transparent polydimethylsiloxane (PDMS) blocks. The PDMS blocks with slots and microchannels are molded from 3D printed plastic molds. The distance between IEMs is 2 mm with 0.2 mm height and 10 mm length for visualization experiments (Fig. 2), and with 0.6 mm height and 20 mm length for CU/power analysis (Figs 3, 4, 5). Fumasep® FTAM-E, FTCM-E (FuMA-Tech GmbH, Germany), and carbon paper (Fuel Cell Store, Inc., Boulder, CO) are used as AEMs, CEMs, and electrodes respectively. On the commercial microscope (Olympus, IX-71), in 10 mM NaCl, LiCl, and KCl solutions, fluorescent dye −0.78 μM Alexa Fluor 488 (Invitrogen, Carlsbad, CA) or 5 μM rhodamine 6 g (R6G)(Sigma Aldrich, St. Louis, MO) −is added to visualize ICP phenomenon. Here, we used positively charged R6G for AEMs-only device (ICP − AEMs) and negatively charged Alexa Fluor 488 for CEMs-only device (ICP − CEMs) and standard ED, respectively. These dyes were selected to visualize the trapped ions between two identical IEMs. Dye concentrations are relatively low, so they do not contribute to the current flow yet can still represent local ion concentrations^47^. A thermoelectrically cooled charge-coupled device (CCD) camera (Hamamatsu Co., Japan) recorded the fluorescent image sequences, and the images were analyzed by ImageJ (NIH, Bethesda, MD). The pressure-driven flow was generated by syringe pump (Harvard apparatus, PHD 2200), resulting the parabolic velocity profile of Hagen-Poiseuille flow between the membranes. The pumping energy is negligibly small compared to the electric energy (SI Section 5). Voltage responses are measured during 300 sec and averaged at a constant applied current with current-voltage source measurement unit (Keithley 236, Keithley Instruments, Inc., Cleveland, OH); this averaged data is used to calculate desalination metrics, and we did not consider its fluctuation which is negligibly small. To quantify the desalting performance, we traced the conductivity of desalted and brine flows directly by mounting a flow-through conductivity probe (Microelectrode, Inc, Bedford, NH) at the end of the desalted/brine channels. The probe is connected to a benchtop conductivity meter (VWR symphony conductivity meter, VWR International LLC., Atlanta, GA).

### Numerical Simulation

ICP desalination system is governed by Poisson-Nernst-Planck (PNP) equations for ion transport and by Navier-Stokes (NS) equations for fluid flows^7^. The equations were discretized using implicit finite volume method. Then, the discretized equations are solved accurately using the GMRES method^48^. The sets of PNP equations are solved iteratively until solution converged. Due to the strongly coupled equations, especially near the membrane surface, they are solved simultaneously using Newton-Raphson method. Obtained ion concentration and electric potential are then used to calculate the electrical volume force, which is involved in solving NS equations. Obtained velocity field is used for the next PNP-NS iteration. In this simulation model, the membrane is assumed to have an ideal perm-selectivity; that is, the current is carried solely by counter-ions, and zero co-ion flux is enforced^16^,^18^. Detail simulation method is described in SI Section 3. The simulation model has 60 μm width, 150 μm length, and a unit height 1 m with 7.9 mm/s (*U*_HP_ = 800*U*_0_, *U*_0_ = 9.89 μm/s) as an averaged velocity of Hagen-Poiseuille flow.

## Additional Information

**How to cite this article**: Kwak, R. *et al.* Enhanced Salt Removal by Unipolar Ion Conduction in Ion Concentration Polarization Desalination. *Sci. Rep.* **6**, 25349; doi: 10.1038/srep25349 (2016).

## Supplementary Material

Supplementary Information
