# Supplementary material for: Enhanced Salt Removal by Unipolar Ion Conduction in Ion Concentration Polarization Desalination
Source: Sci Rep. 2016 May 9;6:25349. doi: 10.1038/srep25349 (PMC4860715; doi:10.1038/srep25349)
Supplement: Supplementary Information [file srep25349-s1.pdf]

SUPPLEMENTARY INFORMATION:

Enhanced Salt Removal by Unipolar Ion Conduction in Ion Concentration Polarization Desalination

Rhokyun Kwak, Van Sang Pham, Bumjoo Kim, Lan Chen and Jongyoon Han

1. Theoretical Current Utilization (CU) and Energy per Ion Removal (EPIR): Control Volume Analysis

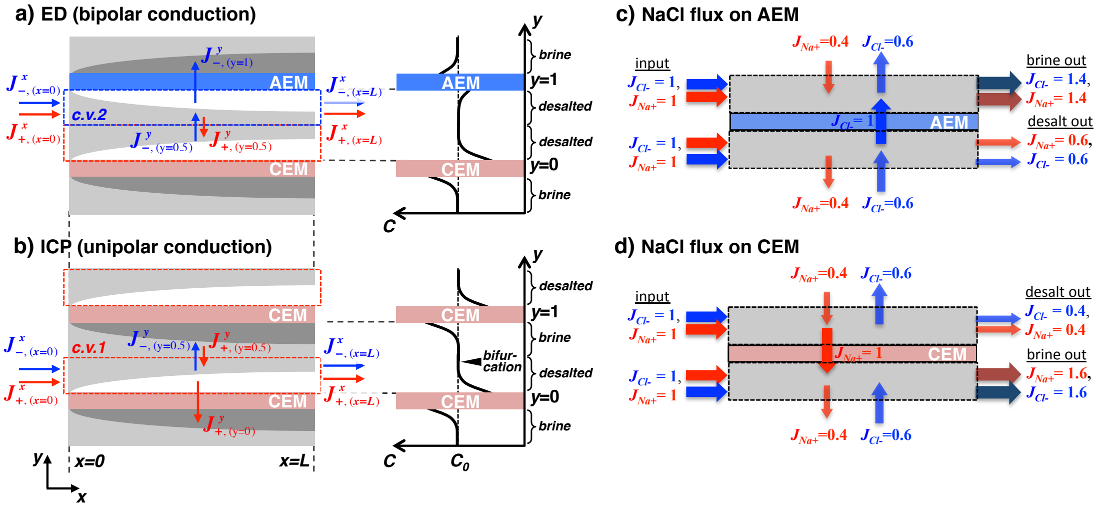

SI Figure 1 | Control volume setting and NaCl flux matching model. a-b, Schematic diagrams of one membrane pair (bipolar ED (**a**) and unipolar ICP (**b**)) with control volume (c.v.) including one desalted flow on the CEM or the AEM (dotted boxes). Concentration profiles **a,** ED and **b,** ICP system are plotted on the right side. Blue and red arrows represent the fluxes J of anion (subscript −) and cation (subscript +) at the four c.v. boundaries. Ion depletion zones occur at the anodic side of CEMs and at the cathodic side of AEMs (white regions (**a-b**)); and *vice versa* for ion enrichment zones (dark gray regions (**a-b**)). **c-d**, Na^+ and Cl^- flux matching in each c.v.. Blue and red arrows indicate the flux of Cl^- and Na^+, respectively. The color / thickness of arrows represent the magnitude of fluxes qualitatively.

For experimental test, we select one membrane pair of ED or ICP systems (N=1), as described in Fig.1a-b, including two membranes and corresponding two desalted flows and two brine flows (SI Fig. 1a-b). Here, according to the definition of CU, we can calculate it theoretically as;

$$CU = \frac{\text{ion removal}}{\text{ion current}} = \frac{\text{ion flux at inlet - ion flux at outlet}}{\text{ion flux through membranes}}. \qquad \text{(SI 1.1)}$$

For control volume analysis, we set control volumes (c.v.) separately for each depletion zone near the membranes; one on CEMs (c.v.1, red dotted boxes) and another on AEMs (c.v.2, blue dotted box)(SI Fig. 1a-b). Each c.v. has two boundaries facing x-direction (at x=0 and x=L) and the other two boundaries facing y-direction at

y=0 and y=0.5 for c.v.1 (or at y=0.5 and y=1 for c.v.2). Here, the ion fluxes normal to these boundaries are defined as $J^{k}_{i,(boundary\ location)}$, where i is the ion species (+: cation, −: anion) and k is the direction of the flux (x: x-direction, y: y-direction);

$$\begin{aligned} J^{x}_{i,(x=0)} &= \int_{y=0}^{y=0.5} j^{x}_{i,(x=0)}\,dy = \int_{y=0.5}^{y=1} j^{x}_{i,(x=0)}\,dy = \int_{y=1}^{y=1.5} j^{x}_{i,(x=0)}\,dy = \int_{y=1.5}^{y=2} j^{x}_{i,(x=0)}\,dy, \\ J^{x}_{i,(x=L)} &= \int_{y=0}^{y=0.5} j^{x}_{i,(x=L)}\,dy \text{ (for c.v.1) or } \int_{y=0.5}^{y=1} j^{x}_{i,(x=L)}\,dy \text{ (for c.v.2)} \end{aligned} \quad , \text{(SI 1.2)}$$

$$J^{y}_{i,(y=0\text{ or }0.5\text{ or }1)} = \int_{x=0}^{x=L} j^{y}_{i,(y=0\text{ or }0.5\text{ or }1)}\,dx\,, \quad \text{(SI 1.3)}$$

where j is ion flux density at each point. The origin of x-y plane (0,0) is on the left corner of the bottom CEM. The upper CEM or AEM is located at y=1. According to the bulk electroneutrality, the cation flux and anion flux by convection (parabolic pressure driven flow) at the inlet (x=0) and the outlet (x=L) are the same;

$$J^{x}_{+,(x=0)} = J^{x}_{-,(x=0)}\,, \quad \text{(SI 1.4)}$$

$$J^{x}_{+,(x=L)} = J^{x}_{-,(x=outlet)}\,. \quad \text{(SI 1.5)}$$

We assume that ions can be transferred only by convection in x-direction, and IEMs have the perfect perm-selectivity with zero cation (anion) flux through AEM (CEM).

1.1. Standard bipolar ED

Applying Eq. SI 1.1-1.5 for c.v.1 and c.v.2, current utilizations (CU) become;

$$CU_{c.v.1} = \frac{(J^{x}_{+,(x=0)} - J^{x}_{+,(x=L)}\big|_{c.v.1}) + (J^{x}_{-,(x=0)} - J^{x}_{-,(x=L)}\big|_{c.v.1})}{J^{y}_{+,(y=0)}}, \quad \text{(SI 1.6)}$$

$$CU_{c.v.2} = \frac{(J^{x}_{+,(x=0)} - J^{x}_{+,(x=L)}\big|_{c.v.2}) + (J^{x}_{-,(x=0)} - J^{x}_{-,(x=L)}\big|_{c.v.2})}{J^{y}_{-,(y=1)}}. \quad \text{(SI 1.7)}$$

Because the amounts of anion and cation in c.v. should be equal (*i.e.* electroneutrality), under steady state condition, the sum of cation/anion fluxes into and out of c.v. are zero. Here, we have no "ion generation" (*e.g.* water splitting) in c.v.s. From the charge conservation in c.v.1,

$$\begin{cases} J^{x}_{+,(x=0)} - J^{x}_{+,(x=L)}\big|_{c.v.1} = J^{y}_{+,(y=0)} - J^{y}_{+,(y=0.5)} \\ J^{x}_{-,(x=0)} - J^{x}_{-,(x=L)}\big|_{c.v.1} = J^{y}_{-,(y=0.5)} \end{cases}. \quad \text{(SI 1.8)}$$

In c.v.2,

$$\begin{cases} J^{x}_{+,(x=0)} - J^{x}_{+,(x=L)}\Big|_{c.v.2} = J^{y}_{+,(y=0.5)} \\ J^{x}_{-,(x=0)} - J^{x}_{-,(x=L)}\Big|_{c.v.2} = J^{y}_{-,(y=1)} - J^{y}_{-,(y=0.5)} \end{cases} . \quad \text{(SI 1.9)}$$

Substituting charge conservation equations (Eq. SI 1.8-1.9) into Eq. SI 1.6-1.7,

$$CU_{c.v.1} = \frac{2J^{y}_{-,(y=0.5)}}{J^{y}_{+,(y=0)}} , \quad \text{(SI 1.10)}$$

$$CU_{c.v.2} = \frac{2J^{y}_{+,(y=0.5)}}{J^{y}_{-,(y=1)}} . \quad \text{(SI 1.11)}$$

From Eq. SI 1.4-1.5 and Eq. SI 1.8-1.9, we also obtain

$$J^{y}_{+,(y=0)} = J^{y}_{+,(y=0.5)} + J^{y}_{-,(y=0.5)} = J^{y}_{-,(y=1)} . \quad \text{(SI 1.12)}$$

For one membrane pair of ED, combining c.v.1 and c.v.2, the CU_{ED} is

$$CU_{ED} = \frac{1}{2}\left(CU_{c.v.1} + CU_{c.v.2}\right) = \frac{J^{y}_{+,(y=0.5)} + J^{y}_{-,(y=0.5)}}{J^{y}_{+,(y=0)}} = 1. \quad \text{(SI 1.13)}$$

At the middle of the channel (y=0.5), if we assume only electromigration is a dominant transport mechanism in y-direction (negligible y-directional convection and diffusion),

$$\frac{J^{y}_{-,(y=0.5)}}{J^{y}_{+,(y=0.5)}} = \frac{D_{-}}{D_{+}} . \quad \text{(SI 1.14)}$$

In other word, this assumption indicates that the fast pressure driven flow prevents the overlap of the depletion boundaries. Substituting Eq. SI 1.14 and arranging Eq. SI 1.13 again, CU_{ED} should be always 1, regardless of the kind of electrolytes we have;

$$CU_{\mathrm{ED}} = \frac{D_{-} + D_{+}}{D_{-} + D_{+}} = 1 \quad \text{(SI 1.15)}$$

1.2. Unipolar ICP desalination

In two identical c.v.s in ICP-CEMs (SI Fig. 1b), CU calculation and charge conservation equations are the same with bipolar ED case. One difference is that the current efficiencies of two c.v.1s are the same, resulting;

$$CU_{c.v.1} = CU_{c.v.1'} = \frac{2J^{y}_{-,(y=0.5)}}{J^{y}_{+,(y=0)}} = \frac{2J^{y}_{-,(y=1.5)}}{J^{y}_{+,(y=1)}} , \quad \text{(SI 1.16)}$$

where $J^{y}_{-,(y=0.5)} = J^{y}_{-,(y=1.5)}$ and $J^{y}_{+,(y=0)} = J^{y}_{+,(y=1)}$. Then, the CU of ICP-CEMs is

$$CU_{ICP} = \left.\frac{2D_-}{D_- + D_+}\right|_{ICP-CEMs}. \quad \text{(SI 1.17)}$$

According to the same analogy, the CU of ICP-AEMs is

$$CU_{ICP} = \left.\frac{2D_+}{D_- + D_+}\right|_{ICP-AEMs}. \quad \text{(SI 1.18)}$$

As noticed in Eq. SI 1.10-1.11 and Eq. SI 1.17-1.18, CU values for the two depletion zones are dissimilar on CEM and AEM, therefore has different contributions to overall desalination. The unipolar ICP system can utilize only CEM or AEM depletion zones, instead of both present in ED. For example, with NaCl solution (D_{Na+}=1.33, D_{Cl-}=2.03 [10^{-9} m^2s^{-1}])[1], total ion flux is split into ~ 60 % Cl^- flux and ~40% Na^+ flux at the center (y=0.5) (SI Fig. 1c-d), due to their inherent mobility differences. As a result, from both control volume analysis and flux matching scenario,

$$\begin{aligned} &CU_{\mathrm{ED}} = 1, \\ &CU_{ICP-CEMs} = 1.208, \\ &CU_{ICP-AEMs} = 0.792. \end{aligned} \quad \text{(SI 1.19)}$$

1.3. Extension to multivalent / multicomponent case

The control volume analysis above can be expanded for general electrolytes;

$$CU_{ICP} = \left.\frac{2\sum J_{-,y=0.5}}{\sum J_{+,y=0.5} + \sum J_{-,y=0.5}}\right|_{ICP-CEMs} or \left.\frac{2\sum J_{+,y=0.5}}{\sum J_{+,y=0.5} + \sum J_{-,y=0.5}}\right|_{ICP-AEMs}. \quad \text{(SI 1.20)}$$

From the Einstein relation (D_i=RTv_i, where diffusivity D_i, gas constant R, temperature T, and mobility v_i), the flux by electric field E is $J_i = (FE/RT)z_iD_ic_i$, where z_i is charge number or ion valance, c_i is ion concentration, and F is faraday constant (=9.65×10^4 $C\cdot mol^{-1}$). The subscript i indicates the specific anion or cation. Substituting J_i in Eq. SI 1.20 with the constitutive equation of electroneutrality,

$$\begin{aligned} &CU_{ICP-CEMs} = \frac{2\sum z_-c_-D_-}{\sum z_-c_-D_- + \sum z_+c_+D_+}, \\ &CU_{ICP-AEMs} = \frac{2\sum z_+c_+D_+}{\sum z_-c_-D_- + \sum z_+c_+D_+}, \quad \sum z_-c_- = \sum z_+c_+. \end{aligned} \quad \text{(SI 1.21)}$$

The majority anion in most naturally occurring source waters is chloride ion, which is one of the fast ions. So, most cations found in source waters have smaller mobility than chloride ion, resulting in better CU with CEMs-only device (ICP-CEMs). According to Vermass *et al.*[2], the major salt contents in natural seawater / river water are Na^{+} (202 / 38.7 mM), Ca^{+} (7 / 2.2 mM), Mg^{+} (28 / 3.3 mM), Cl^{-} (242 / 43.9 mM), and SO^{4-} (11 / 2.3 mM). The diffusivities of Na^{+}, Ca^{2+}, Mg^{2+}, Cl^{-}, and SO_4^{2-} are D_{Na^+}=1.334, $D_{Ca^{2+}}$=0.791, $D_{Mg^{2+}}$=0.7, and $D_{SO_4^{2-}}$=1.065 [10^{-9} m^2/s][1, 3]. In seawater case,

$$CU_{ICP-CEMs} = \frac{2\left(1\cdot 242\cdot D_{Cl^-} + 2\cdot 11\cdot D_{SO_4^{2-}}\right)}{1\cdot 242\cdot D_{Cl^-} + 2\cdot 11\cdot D_{SO_4^{2-}} + 1\cdot 202\cdot D_{Na^+} + 2\cdot 28\cdot D_{Mg^{2+}} + 2\cdot 7\cdot D_{Ca^{2+}}} \approx 1.234, \quad \text{(SI 1.22)}$$

$$CU_{ICP-AEMs} = 2 - CU_{ICP-CEMs} \approx 0.766\,. \quad \text{(SI 1.23)}$$

Next, in river water case,

$$CU_{ICP-CEMs} = \frac{2\left(1\cdot 43.9\cdot D_{Cl^-} + 2\cdot 2.3\cdot D_{SO_4^{2-}}\right)}{1\cdot 43.9\cdot D_{Cl^-} + 2\cdot 2.3\cdot D_{SO_4^{2-}} + 1\cdot 38.7\cdot D_{Na^+} + 2\cdot 3.3\cdot D_{Mg^{2+}} + 2\cdot 2.2\cdot D_{Ca^{2+}}} \approx 1.244, \quad \text{(SI 1.24)}$$

$$CU_{ICP-AEMs} = 2 - CU_{ICP-CEMs} \approx 0.756\,. \quad \text{(SI 1.25)}$$

For the above two natural waters, ICP-CEMs allows CU enhancement by ~24% (EPIR/V^* is decreased by ~20%). Even for special cases with slower anion (*e.g.* mixture of $NaHCO_3$ and NaCl, $D_{HCO^{3-}}$=1.185 [10^{-9} m^2/s][3]), ICP-CEMs would be better if $c_{Cl-} > c_{HCO3-}$. If $c_{Cl-} < c_{HCO3-}$, desalinating with ICP-AEMs will have higher CE than ICP-CEMs.

For simple multivalent binary electrolytes (*e.g.* $CaCl_2$, $MgCl_2$), The Eq. SI 1.21 becomes

$$CU = \left.\frac{2z_-c_-D_-}{z_-c_-D_- + z_+c_+D_+}\right|_{ICP-CEMs} = \left.\frac{2z_+c_+D_+}{z_-c_-D_- + z_+c_+D_+}\right|_{ICP-AEMs}. \quad \text{(SI 1.26)}$$

Substituting the electroneuatrality equation, $z_+c_+ = z_-c_-$,

$$CU = \left.\frac{2D_-}{D_- + D_+}\right|_{ICP-CEMs} = \left.\frac{2D_+}{D_- + D_+}\right|_{ICP-AEMs} \quad \text{(SI 1.27)}$$

As can be seen, CU values are independent with ion valence z. CU is only depending on ions' diffusivity.

1.4. Energy Efficiency: Energy per Ion Removal (EPIR)

To compare different desalination devices, energy consumption has been frequently measured. In electrochemical desalination systems, energy consumption is evaluated by the electrical power consumption (=multiplication of current I and voltage V) divided by the flow rate of desalted water Q_{desalted};

$$\text{Energy consumption } = \frac{IV}{Q_{desalted}}[Wh/L]. \quad \text{(SI 1.28)}$$

While energy consumption is a widely-used metric determining the economic viability of the desalination technique, we also consider energy consumption to remove a single pair of ions (*i.e.* ion valence)(*e.g.* 1 for NaCl), *i.e.* energy per ion removal (EPIR), which can be obtained by dividing energy consumption by the amount of removed ions, and then non-dimensionalizing the value by thermal energy $k_B T$ (=2.479 kJ/mol):

$$\text{Energy per ion removal } = \frac{IV/Q_{desalted}}{zk_B T(C_0 - C_{desalted})}, \quad \text{(SI 1.29)}$$

where z is ion valance (averaged ion valance for multivalent / multicomponent cases), C_0 is the initial ion concentration, C_{desalted} is the ion concentration of desalted flow. EPIR is a parameter representing how efficiently energy is consumed to separate ions by combining the concept of energy consumption and salt removal ratio; therefore, EPIR represents the inherent energy efficiency of a given desalination process. As described in Eq. 2, the Eq. SI 1.29, EPIR can be expressed with CU and non-dimentionalized voltage V^*;

$$\text{EPIR} = \frac{IV/Q_{desalted}}{zk_B T(C_0 - C_{desalted})} = \frac{V^*}{CU}, \quad V^* = \frac{FV}{k_B T}. \quad \text{(SI 1.30)}$$

F indicate Faraday's constant (=9.65×10^4 C·mol^{-1}) respectively. In the case of standard bipolar ED, CU is always one, resulting EPIR/V^* =1; however, in unipolar

ICP, EPIR/V* also can be shifted to the opposite direction of the CU shift. For NaCl solution, substituting CU values (Eq. SI 1.19) in Eq. SI 1.30,

$$\begin{aligned} &\mathrm{EPIR} / V^{*}_{\mathrm{ED}} = 1, \\ &\mathrm{EPIR} / V^{*}_{ICP-CEMs} = 0.827, \\ &\mathrm{EPIR} / V^{*}_{ICP-AEMs} = 1.263. \end{aligned} \qquad \text{(SI 1.31)}$$

Also, in the case of natural seawater and river water, from Eq. SI 1.22-1.25,

$$\begin{aligned} &\text{seawater:}\quad \mathrm{EPIR} / V^{*}_{ICP-CEMs} = 0.810, \\ &\text{river water: } \mathrm{EPIR} / V^{*}_{ICP-CEMs} = 0.804. \end{aligned} \qquad \text{(SI 1.32)}$$

1.5. Definition of Current Utilization (CU)

Some ED literatures define the current efficiency (CE) per each pair of membranes[4, 5], while others define it per each 'cell' (one desalting and one brine flow pairs). For ED process, those two definitions are equivalent (Fig. 1a and SI Fig. 2a). Here, 1 desalting and 1 brine flow are generated for each pair of membranes (one AEM and one CEM). However, for ICP process, it is clear that 2 desalting flows and 2 brine flows are generated for each pair of membranes (two CEMs)(Fig. 1b and SI Fig. 2b).

In terms of comparing the ideality of the membrane process in different systems (which is the role of the CE), it is important to ensure fair comparison between ED and ICP systems, on the basis of equal functionality: more specifically, systems with the same number of membranes and the same output flow rates. Therefore, in this work, we define CE (which we call as CU) per each pair of membranes. In other words, unit cell (N=1) is defined for each pair of membranes, whether they are AEMs or CEMs. This definition can be applied to not just ED but ICP, as well as other electrochemical processes such as capacitive deionization[6] and electrolyzer for chemical productions[7, 8]. This CU definition is, therefore, a more general form that is applicable to various electrochemical systems.

Even sticking on the CE definition with one desalting flow, we obtain the same result that ICP-CEMs has better salt removal than ED. Approximately, rearranging the equation (1);

$$CE \sim \frac{Q_{desalted}\Delta C}{NI}, \qquad \text{(SI 1.33)}$$

where ΔC is the amount of salt removal, ΔC=C_0-$C_{desalted}$. Here, one ICP cell is also defined with one desalted flow and one brine flow (SI Fig. 2b). Then, in this case, the desalted flow rate in a unit ICP-CEMs cell is $Q/2$, while that in a unit ED cell is Q. As a result, as the desalted flow rate is halved in Eq. SI 1.33 (compared to CU), CE for ICP-CEMs is ~0.6 in NaCl solution; while still CE=1 for ED. Although CE of ICP-CEMs is lower than ED, we have the doubled number of desalted flows under the same functionality (same number of membranes). As can be seen in SI Fig. 2, N=2 for ED and N=4 for ICP-CEMs. Therefore, with the same functionality, the total amount of salt removal ΔC of ICP-CEMs and ED are;

$$\Delta C \sim \frac{NI \cdot CE}{Q_{desalted}},$$
$$\Delta C\big|_{ICP-CEMs} = \frac{4I \cdot 0.6}{2Q} = 1.2I/Q, \qquad \text{(SI 1.34)}$$
$$\Delta C\big|_{ED} = \frac{2I \cdot 1}{2Q} = I/Q,$$

resulting ~20% enhancement of total salt removal with ICP-CEMs; this is directly represented by the definition of CU in the main text.

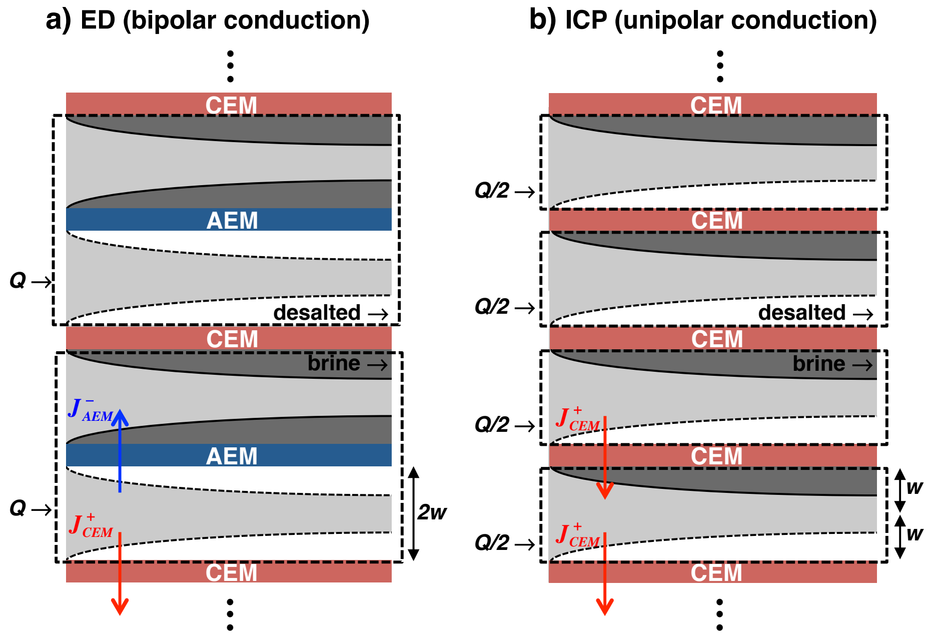

SI Figure 2 | Schematic diagrams of a) ED and b) ICP-CEMs with the same membrane numbers. One black dotted box includes one desalting flow; Q (flow width: 2w) and $Q/2$ (flow width: w) indicate the desalted flow rates.

2. Differential Equation Analysis in ICP Desalination with Linear ICP Model

2.1. Governing equations

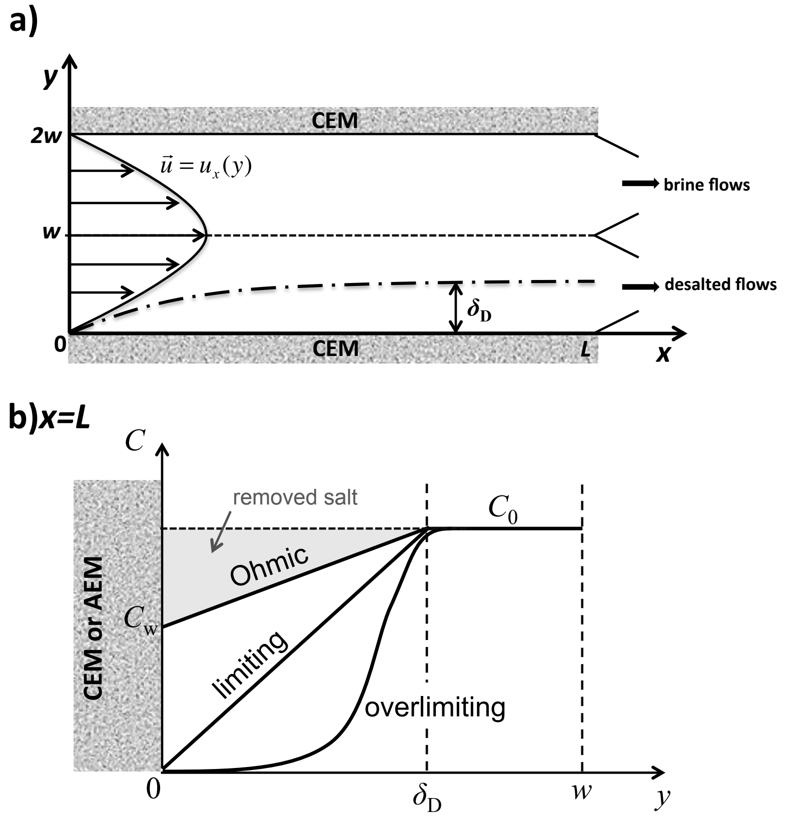

SI Figure 3 | Analysis model of ICP-CEMs. a, Schematic figure and **b,** ion concentration profile at the outlet ($x=L$). The channel width of brine and desalted flows is w.

Physicochemical hydrodynamic systems including ED typically defined by three governing equations: Nernst-Plank equation, Poisson equation, and Navier-Stokes equations, which describe ion transport, ion's distribution under electric field, and fluid motion, respectively. Here, without chemical reactions, the governing equations are;

$$\vec{J} = -F\sum_i z_i D_i \nabla c_i - F^2 \nabla\varphi \sum_i M_i z_i^2 c_i + F\vec{u}\sum_i z_i c_i \,, \tag{SI 2.1}$$

$$\sum_i z_i F c_i = \rho_e = -\nabla \cdot (\varepsilon \nabla \varphi)\,, \tag{SI 2.2}$$

$$\rho\left(\frac{\partial \vec{u}}{\partial t} + \vec{u}\cdot\nabla\vec{u}\right) = -\nabla P + \mu\nabla^2\vec{u} - \rho_e \nabla\varphi \,. \tag{SI 2.3}$$

All symbols used here described in SI Table 1. From mass continuity of species i, Eq. SI 2.1 becomes;

$$\frac{\partial c_i}{\partial t} + \vec{u} \cdot \nabla c_i = \nabla \cdot \left(D_i \nabla c_i + M_i z_i F c_i \nabla \varphi \right) \qquad \text{(SI 2.4)}$$

For dilute binary electrolyte, from Eq. SI 2.2 and electroneutrality of bulk solutions (ρ_e=0), $z_+c_+= -z_-c_-=c$. By adding or subtracting an equation set from Eq. SI 2.4 for cation and anion, we obtain;

$$0 = (D_+ - D_-)\nabla^2 c + (D_+ + D_-)\frac{F^2}{RT}\nabla \cdot (c\nabla\varphi)\,, \qquad \text{(SI 2.5)}$$

$$2\left(\frac{\partial c}{\partial t} + \vec{u} \cdot \nabla c\right) = (D_+ + D_-)\nabla^2 c + (D_+ - D_-)\frac{F^2}{RT}\nabla \cdot (c\nabla\varphi)\,. \qquad \text{(SI 2.6)}$$

Substituting Eq. SI 2.5 to Eq. SI 2.6, we can obtain conventional convective-diffusion equation with effective diffusivity D_{eff} (Eq. 3.4.12 in Probstein, R.F.[3]);

$$\frac{\partial c}{\partial t} + \vec{u} \cdot \nabla c = D_{eff}\nabla^2 c, \quad D_{eff} = \frac{2D_+D_-}{D_+ + D_-} \qquad \text{(SI 2.7)}$$

In 2-D ICP-CEMs model described in SI Fig. 3a, we assume no vertical fluid motion (u_y=0), negligible horizontal electromigration (E_x=0) and diffusion. Then, from Nernst-Plank equation (Eq. SI 2.1) and Poisson-Nernst-Plank equation (Eq. SI 2.7), substituting Einstein relation (D_i=RTM_i),

$$\frac{\partial c}{\partial t} = D_{eff}\frac{\partial^2 c}{\partial y^2} \text{ and } D_{eff}\frac{\partial^2 c}{\partial y^2} \sim u_x(y)\frac{\partial c}{\partial x}, \quad D_{eff} = \frac{2D_+D_-}{D_+ + D_-}\,, \qquad \text{(SI 2.8)}$$

$$J_{+,y} = -FD_+\frac{\partial c}{\partial y} - \frac{F^2}{RT}D_+c\frac{\partial \varphi}{\partial y}\,, \qquad \text{(SI 2.9)}$$

$$J_{-,y} = -FD_-\frac{\partial c}{\partial y} + \frac{F^2}{RT}D_-c\frac{\partial \varphi}{\partial y}\,, \qquad \text{(SI 2.10)}$$

where the subscript i and y indicate the species i and the direction of flux, respectively. Lastly, the known solution of Eq. SI 2.3 for parabolic Hagen-Poiseuille flow between two CEMs is;

$$u_x(y) = -\frac{y}{\mu}\frac{dP}{dx}\left(w - \frac{y}{2}\right), \qquad \text{(SI 2.11)}$$

with no change of velocity profile ($\partial u_x/\partial x$=0).

2.2. Analytic solution for linear ICP model

If we assume the diffusive boundary layer thickness δ_D is smaller than the half of the channel width, $\delta_D<w$ (SI Fig. 3a), we can solve Eq. SI 2.8 with approximation of linear velocity profile, resulting (Eq. 6.2.12 in Probstein, R.F.[3]);

$$u_x(y) \sim y \left.\frac{\partial u_x}{\partial y}\right|_{y=0} = \frac{3U_{HP}}{w} y, \qquad \text{(SI 2.12)}$$

$$\frac{\delta_D}{w} \sim \left(\frac{D_{eff}}{U_{HP} w}\right)^{1/3} \left(\frac{x}{w}\right)^{1/3}, \qquad \text{(SI 2.13)}$$

where U_{HP} is the average fluid velocity of Hagen-Poiseuille flow. From Eq. SI. 2.9-2.10, with ideal CEM (perfect perm-selectivity), the current flux densities of cation and anion become;

$$J_{-,y=0} = -FD_- \left.\frac{\partial c}{\partial y}\right|_{y=0} + \frac{F^2}{RT} D_- c \left.\frac{\partial \varphi}{\partial y}\right|_{y=0} = 0, \qquad \text{(SI 2.14)}$$

$$J_{+,y=0} = -FD_+ \left.\frac{\partial c}{\partial y}\right|_{y=0} - \frac{F^2}{RT} D_+ c \left.\frac{\partial \varphi}{\partial y}\right|_{y=0} = -2FD_+ \left.\frac{\partial c}{\partial y}\right|_{y=0}. \qquad \text{(SI 2.15)}$$

As can be seen in SI Fig. 3b, concentration profile in the boundary layer is linear in Ohmic and limiting regimes[3]. Then, by substituting Eq. SI 2.13, the concentration gradient in linear ICP is;

$$\left.\frac{\partial c}{\partial y}\right|_{0\le y<\delta_D} \sim \frac{c_0 - c_w}{\delta_D} \sim \frac{c_0 - c_w}{c_0} \cdot c_0 \left(\frac{U_{HP}}{D_{eff} w x}\right)^{1/3}, \qquad \text{(SI 2.16)}$$

$$c(x,y)\big|_{0\le y<\delta_D} \approx \frac{c_0 - c_w}{c_0} \cdot c_0 \left(\frac{U_{HP}}{D_{eff} w x}\right)^{1/3} y + c_w. \qquad \text{(SI 2.17)}$$

Now, with concentration profile (Eq. SI 2.17) and velocity profile (Eq. SI 2.12), we can calculate the current through the CEM (I_+ at y=0 or 1) and salt removal ratio. First, the current is the integration of current density $J_{+,y=0}$ along the CEM;

$$\begin{aligned} I_+ &= \int_{x=0}^{x=L} J_{+,y=0} h\, dx = \int_{x=0}^{x=L} -2FD_+ \left.\frac{\partial c}{\partial y}\right|_{y=0} h\, dx \\ &= 2FD_+ h (c_0 - c_w) \int_{x=0}^{x=L} \left(\frac{U_{HP}}{D_{eff} w x}\right)^{1/3} dx \end{aligned}. \qquad \text{(SI 2.18)}$$

As a result,

$$I_+ = \frac{c_0 - c_w}{c_0} \cdot \frac{3c_0 FhD_+ U_{HP}^{1/3} L^{2/3}}{D_{eff}^{1/3} w^{1/3}} . \tag{SI 2.19}$$

Next, salt removal ratio can be defined as;

$$\frac{C_0 - C_{desalted}}{C_0} = \frac{\int_{y=0}^{y=w} (c_0 - c(y)) u_x(y)\, dy}{\int_{y=0}^{y=w} c_0 u_x(y)\, dy} . \tag{SI 2.20}$$

Substituting concentration profile (Eq. SI 2.17) and velocity profile (Eq. SI 2.12), the salt removal ratio is;

$$\begin{aligned} \frac{C_0 - C_{desalted}}{C_0} &= \left. \frac{\int_{y=0}^{y=\delta_D} (c_0 - c_w - \frac{c_0 - c_w}{\delta_D} y) \cdot \frac{3U_{HP}}{w} y\, dy}{\int_{y=0}^{y=w} c_0 \cdot \frac{3U_{HP}}{w} y\, dy} \right|_{x=L} . \\ &= \left. \frac{c_0 - c_w}{c_0} \cdot \left(\frac{\delta_D}{w} \right)^2 \right|_{x=L} \end{aligned} \tag{SI 2.21}$$

Substituting the thickness of boundary layer (Eq. SI. 2.13),

$$\frac{C_0 - C_{desalted}}{C_0} = \frac{c_0 - c_w}{c_0} \cdot \frac{L^{2/3} D_{eff}^{2/3}}{U_{HP}^{2/3} w^{4/3}} \tag{SI 2.22}$$

Now, we know the salt removal ratio and the current of unipolar ICP desalination with CEMs (ICP-CEMs). Substituting Eq. SI 2.19 and Eq. SI 2.22 to the equation for CU (Eq. 1), we can calculate the CU of ICP-CEMs;

$$\begin{aligned} CU &= \frac{zFQ_{desalted}(C_0 - C_{desalted})}{NI_+}, \quad Q_{desalted} = whU_{HP} \\ &\approx FwhU_{HP} c_0 \cdot \frac{c_0 - c_w}{c_0} \cdot \frac{L^{2/3} D_{eff}^{2/3}}{U_{HP}^{2/3} w^{4/3}} \left(\frac{c_0 - c_w}{c_0} \cdot \frac{c_0 FhD_+ U_{HP}^{1/3} L^{2/3}}{D_{eff}^{1/3} w^{1/3}} \right)^{-1}, \end{aligned} \tag{SI 2.23}$$

resulting,

$$CU = D_{eff} / D_+ = \frac{2D_-}{D_+ + D_-} . \tag{SI 2.24}$$

The derivation from full governing equations gives us the same result with the control volume analysis. The CU of unipolar ICP system depends only ions' diffusivities, regardless any geometric parameters and operating conditions. For ICP-AEMs, according to the similar analogy, CU=D_{eff}/D_- = 2 $D_+/(D_+ + D_-)$.

2.3. Source of CU shift in Ohmic and limiting current regimes

In Ohmic regime, according to the Eq. SI 2.19 and Eq. SI 2.22, the current in ICP-CEMs is proportional to $\sim(c_0\text{-}c_w)/c_0\cdot D_+D_{eff}^{-1/3}$ ($\sim(c_0\text{-}c_w)/c_0\cdot D_-D_{eff}^{-1/3}$ in ICP-AEMs) and the salt removal ratio is proportional to $\sim(c_0\text{-}c_w)/c_0\cdot D_{eff}^{2/3}$ in Ohmic regime (V=6 V_0, V_0=25.85mV)(SI Fig. 4d-e). Here, from Poisson-Boltzmann equation, ion concentration at the membrane c_w is linearly proportional to the voltage drop in the boundary layer V_{δ_D} (SI Fig. 4c);

$$\frac{c_0 - c_w}{c_0} = 1 - \exp\left(\frac{zFV_{\delta_D}}{k_B T}\right) \sim V^*_{\delta_D}, \quad V^*_{\delta_D} = \frac{zFV_{\delta_D}}{k_B T}. \tag{SI 2.25}$$

As can be seen in SI Fig. 4a-b, the concentration profiles of ICP-CEMs and ICP-AEMs are all different according to the electrolytes. This indicates the resistances of bulk region (outside of the boundary layer) are different, even for ICP-CEMs and ICP-AEMs in the same electrolyte, resulting different $V^*_{\delta_D}$ and c_w. Therefore, combining the differences of the electric resistance, corresponding concentration profile, and salt removal ratio, CU is shifted according to systems and electrolytes in Ohmic regime.

In limiting regime, the equations become simpler. As the local ion concentration at the membrane c_w goes zero, the current and salt removal ratio are

$$I_+ = \frac{c_0 FhD_+ U_{HP}^{1/3} L^{2/3}}{D_{eff}^{1/3} w^{1/3}} \sim D_+ / D_{eff}^{1/3}, \tag{SI 2.26}$$

$$\frac{C_0 - C_{desalted}}{C_0} = \frac{L^{2/3} D_{eff}^{2/3}}{U_{HP}^{2/3} w^{4/3}} \sim D_{eff}^{2/3}. \tag{SI 2.27}$$

In the case of ICP-AEMs, the current I_- is $\sim D_- / D_{eff}^{1/3}$ and salt removal ratio is $\sim D_{eff}^{2/3}$. As a result, if in the same electrolyte, ICP-CEMs and ICP-AEMs have the exactly same concentration profiles (SI Fig. 5a-b). In this limiting regime, between CEMs-only and AEMs-only systems, CU shift occurs only by the ion flux difference sliding the same concentration gradient.

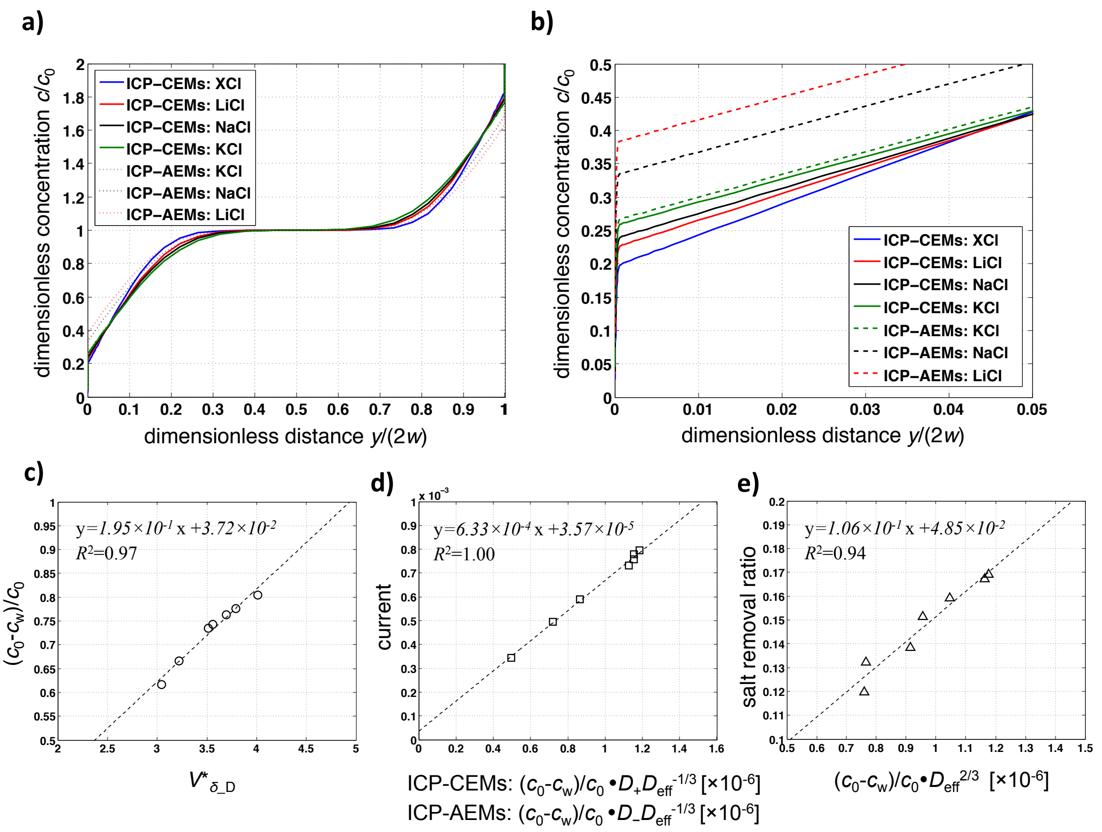

SI Figure 4 | Unipolar ICP desalination in Ohmic regime (simulation model at V=6V_0). a, Dimensionless concentration profile at the outlet (x=L), and **b,** enlarged imaged near the membrane. **c,** The ion concentration at the membrane (y=5×10^{-4}), **d,** current, and **e,** salt removal ratio are plotted against the scaling parameters described by Eq. SI. 2.16-2.25. The non-dimensional voltage drop in diffusive boundary layer $V^*_{\delta_D}$ is calculated by subtracting the voltage at y=0.3 from 6V_0.

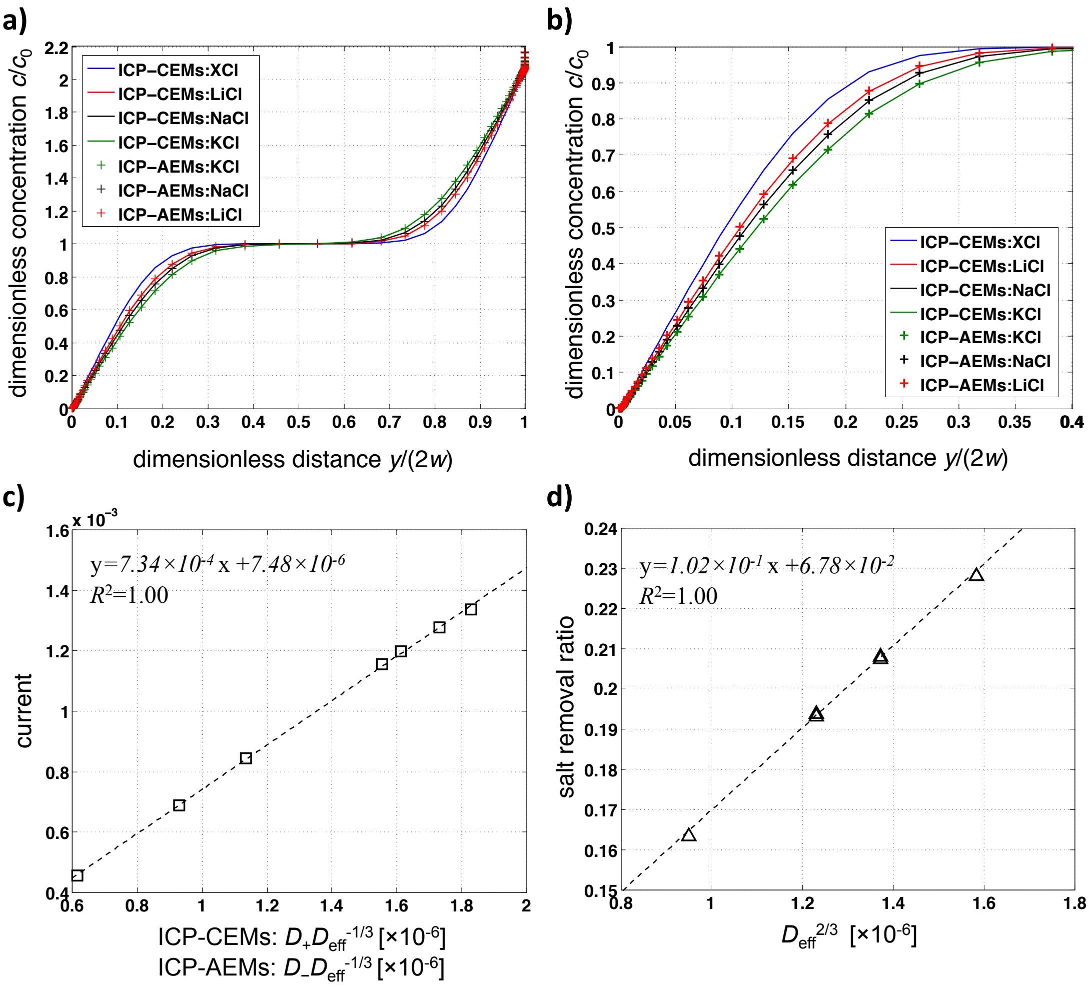

SI Figure 5│ Unipolar ICP desalination in Limiting regime (simulation model at V=20V_0). a, Dimensionless concentration profile at the outlet (x=L), and **b,** enlarged imaged near the membrane. **c,** current (*i.e.* ion flux through the membrane at y=0), and **d,** salt removal ratio are plotted against the scaling parameters described by Eq. SI. 2.26-2.27.

SI Table 1 | List of symbols with units.

Symbol	Description	Unit
$\vec{J}$:	current density	[A m^{-2}]
F:	Faraday's constant (= 9.65 × 10^4)	[C mol^{-1}]
z_i:	charge number of the species *i*	
D_i:	diffusivity of the species *i*	[m^2/s]
c_i:	local molar concentration of the species *i*	[mol m^{-3}]
C_0:	initial ion concentration of electrolyte	[mol m^{-3}]
$C_{desalted}$:	ion concentration of desalted flow	[mol m^{-3}]
φ:	electrostatic potential	[V]
M_i:	mobility of the species *i*	[mol s kg^{-1}]
u:	fluid velocity	[m/s]
ρ_e:	free charge density	[C m^{-3}]
ε:	permittivity	[C V^{-1} m^{-1}]
ρ:	fluid density	[kg m^{-3}]
t:	time	[sec]
μ:	fluid viscosity	[kg m^{-1} s^{-1}]
P:	pressure	[kg m^{-1} s^{-2}]
R:	gas constant	[J mol^{-1}K^{-1}]
T:	temperature	[K]
$\vec{E}$:	electric field	[V/m]
k_B:	Boltzmann constant (=1.38 × 10^{-23})	[m^{2}kg s^{-2}K^{-1}]
h:	channel height of the system	[m]
w:	channel width of the system	[m]
U_{HP}:	average flow velocity	[m/s]

3. Visualization of ICP Phenomenon in Simulation

3.1. Simulation setup (this section is mostly adopted from Kwak *et.al.*[9])

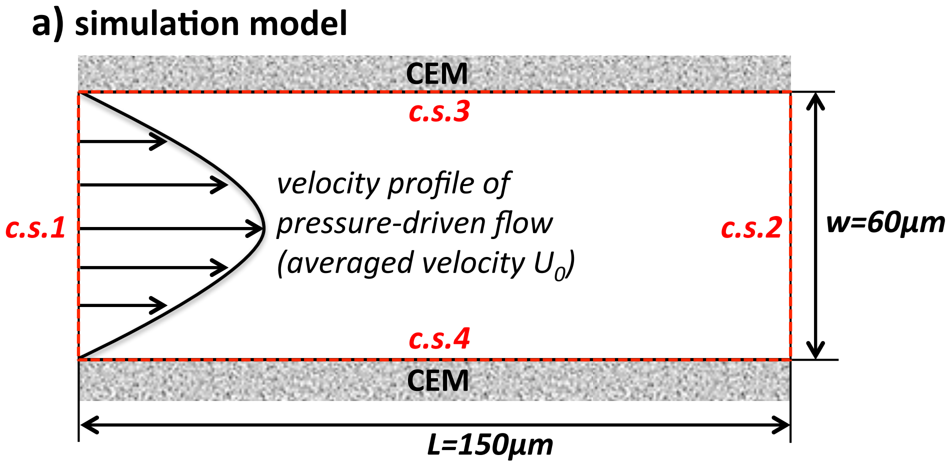

b) boundary conditions

c.s.1 (inlet): $C_{\pm} = C_{bulk};\ \dfrac{\partial \Phi}{\partial n} = 0;\ U = U_{HP}$

c.s.2 (outlet): $\dfrac{\partial C_{+}}{\partial n} = \dfrac{\partial C_{n}}{\partial n} = 0;\ \dfrac{\partial \Phi}{\partial n} = 0;\ \dfrac{\partial U}{\partial n} = 0$

c.s.3 & 4 (CEM): $C_{+} = C_{m};\ J_{-} = 0;\ \Phi = 0;\ U = 0$

SI Figure 6 | Schematic of simulation setup and boundary conditions at each control surface (c.s.)

To observe the dynamics of ICP desalination, we consider a simulation model sketched in SI Fig. 6. Here, we calculate the local ion flux, ion concentration and fluid velocity between CEMs (or AEMs) located on top and bottom sides (red dotted box, SI Fig. 6a). The model has 60μm width, 150μm length, and a unit height 1m with 7.9 mm/s (U_{HP}=800U_0, U_0=9.89μm/s) as an averaged velocity of parabolic Hagen-Poiseuille flow. Simulation is performed by solving the full set of governing equations directly, including the Nernst-Planck equations (Eq. SI 3.1-3.2) for ions transport, the Poisson's equation (Eq. SI 3.3-3.4) for the dependence of electric potential field on the ion concentrations, and the Navier-Stokes and fluid continuity equations (Eq. SI 3.5-3.6) for fluid motion in the channel[10]. These equations are given in dimensionless form as follows,

$$\frac{1}{\tilde{\lambda}_D}\frac{\partial \tilde{C}_{\pm}}{\partial \tilde{t}} = -\nabla \cdot \tilde{J}, \qquad \text{(SI 3.1)}$$

$$\tilde{J}_{\pm} = -\tilde{D}_{\pm}(\nabla \tilde{C}_{\pm} + Z_{\pm}\tilde{C}_{\pm}\nabla\tilde{\Phi}) + Pe\tilde{U}\tilde{C}_{\pm}, \qquad \text{(SI 3.2)}$$

$$\tilde{\lambda}_D^2\nabla\cdot(\nabla\tilde{\Phi}) = -\tilde{\rho}_e, \tag{SI 3.3}$$

$$\tilde{\rho}_e = Z_+\tilde{C}_+ + Z_-\tilde{C}_-, \tag{SI 3.4}$$

$$\frac{1}{Sc}\frac{1}{\tilde{\lambda}_D}\frac{\partial\tilde{U}}{\partial\tilde{t}} = -\nabla\tilde{P} + \nabla^2\tilde{U} - \mathrm{Re}(\tilde{U}\cdot\nabla)\tilde{U} - \tilde{\rho}_e\nabla\tilde{\Phi} = 0, \tag{SI 3.5}$$

$$\nabla\cdot\tilde{U} = 0, \tag{SI 3.6}$$

where $\tilde{t} = t/\tau_0$, $\widetilde{C_\pm} = C_\pm/C_0$, $\tilde{\Phi} = \Phi/\Phi_0$, $\tilde{U} = U/U_0$, $\tilde{P} = P/P_0$, are the normalized time, ion concentrations, electric potential, vector of fluid velocity, and pressure, respectively. The corresponding reference values are given as follows.

$$\tau_0 = \frac{l_0^2}{D_0}; C_0 = C_{bulk}; \Phi_0 = \frac{k_B T}{Ze}; U_0 = \frac{\varepsilon\Phi_0^2}{\eta l_0}; P_0 = \frac{\eta U_0}{l_0} \tag{SI 3.7}$$

where $C_{bulk}$is the bulk concentration, $l_0 = w$ $l_0 = w$is the characteristic length scale, $D_0 = (D_+ + D_-)/2$ is average diffusivity, k_B is the Boltzmann constant, T is absolute temperature, e is the elementary charge, Z is ion valence, η is dynamics viscosity of solution, and ε is permittivity. Parameters $\widetilde{D_\pm} = D_\pm/D_0$, $\widetilde{\lambda_\pm} = \lambda_D/l_0$, and $\widetilde{\rho_e} = \rho_e/C_{bulk}$, are normalized diffusion coefficient, Debye length ($\lambda_D = \sqrt{\varepsilon k_B T / 2C_{\mathrm{bulk}} Z^2 e^2}$) and the space charge, respectively. $Pe = U_0 l_0 / D$, $Sc = \eta / \rho D_0$, and $Re = \rho U_0 l_0 / \eta$ are the Pélect number, the Schmidt number, and the Reynolds number, respectively.

The governing equations (Eq. SI 5.1-5.6) are solved numerically using the finite volume method for discretization and Newton-Raphson method for the nonlinear equations generated by discretization of the Poisson-Nernst-Planck equation[11, 12]. The transient terms are treated implicitly using a three-level scheme which is second-order accuracy. Within each time step the Poisson-Nernst-Planck equations (Eq. SI 5.1-5.4) and the Navier-Stokes equations (Eq. SI 5.5-5.6) are solved in an iterative manner. Starting with a velocity field from previous iteration or initial condition, the Poisson-Nernst-Planck equations are solved simultaneously. The potential and ion concentrations obtained are used to calculate electrical body force. The Navier-Stokes equations are then solved with the electrical body force to obtain the velocity field for the next iteration. The process is iterated until convergence is reached. The Navier-Stokes equations are solved by a coupled method which uses

Rhie-Chow interpolation to produce an equation for pressure from the continuity equation[13, 14]. Linear algebraic equations are solved using the PETSc library which implements the Krylov-subspace method[15]. Due to the rapid change of variable near the membranes surface, mesh for the computational domain is strongly refined near the membranes using GMSH[16].

Boundary conditions are also supplied for the closure of the equations. The boundary conditions are given in SI Fig. 6b, where the permselective membranes are assumed to be impermeable to co-ions, and allow counter-ions to pass through at a constant concentration ($C_m=2C_0$); a pressure gradient is applied between the inlet and the outlet to drive the incoming Hagen-Poiseuille flow with a parabolic velocity profile (U_{HP}: average speed). The common no-slip boundary condition for fluid flow is also enforced at the membrane surfaces.

In this computation, the dimensionless Debye length employed is smaller than that of the experiment; however, we did not use the usual simplifying assumption of the thin Debye layer limit. Instead, the ion concentrations, potential and fluid flow at all voltage biases (Ohmic, limiting and overlimiting regimes) were directly obtained by solving the full set of nonlinear and coupled governing equations (Poisson-Nernst-Planck and Navier-Stokes), and with the no-slip boundary conditions. The slip condition (second-kind electroosmosis) introduced by Rubinstein and Zaltzman[17] is only applicable in overlimiting current regime; this methodology is therefore not useful to predict the threshold voltage of transition between limiting and overlimiting regimes. The electrolyte solution is sodium chloride (NaCl), which can fully disassociate into Na^+ and Cl^- ions in the aqueous solution. Parameters used in this study are given in SI Table 2.

SI Table 2 | Parameters used for simulation

Symbol	Description	Value
T:	absolute temperature	300 K
λ_D:	Debye length	3.1 nm
C_0:	bulk concentration	10 mM
Φ_0:	thermal voltage	25.85 mV
U_0:	velocity scale	9.89 μm/s
l_0:	length scale	60 μm

3.2. ICP phenomenon in simulation model

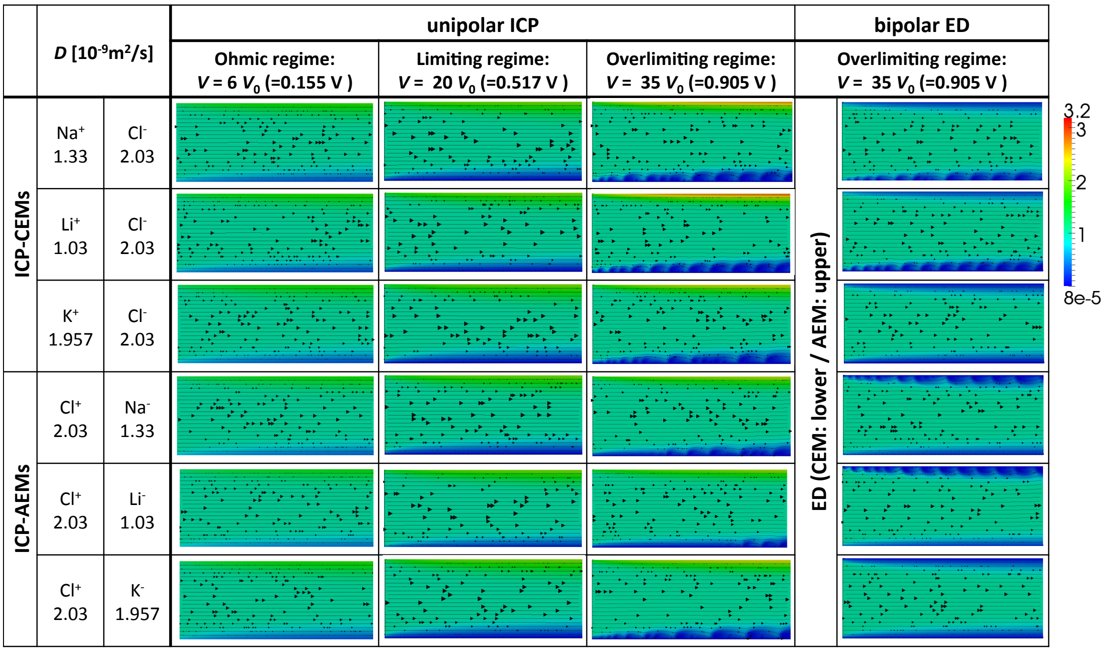

SI Figure 7 | Visualized ICP in simulation model. Color bar indicates the concentration of cation, which is the same with anion's concentration except only on CEMs or AEMs, where at the bottom / top surfaces of the system. Streamlines (black arrows) between CEMs are also visualized. ICP system is simulated at V=6V_0, 20V_0, and 35V_0. ED system is simulated at V=35V_0 (with one CEM (one AEM) on the bottom (top) surface of the system).

ICP phenomenon in ICP and ED systems are visualized with various operating conditions in simulation model (SI Fig. 7). We apply cation / anion diffusivities reversely with CEMs to calculate ICP-AEMs cases, instead of setting AEMs; therefore, under electric field from top to bottom, ion depletion zones show on the bottom membrane.

In SI Figure 8, I-V curves of ICP desalination are also measured in simulation. The saturation of current in limiting regime is clearly observed than that in experiment (Fig.5a). This is possibly due to the rough surface of real IEMs, which can initiate electroconvective vortices more randomly[17, 18]. Here, the shorter plateau length of limiting regime (by fast initiation of vortices) is also well observed at slower counter-ion diffusivity (Fig.5).

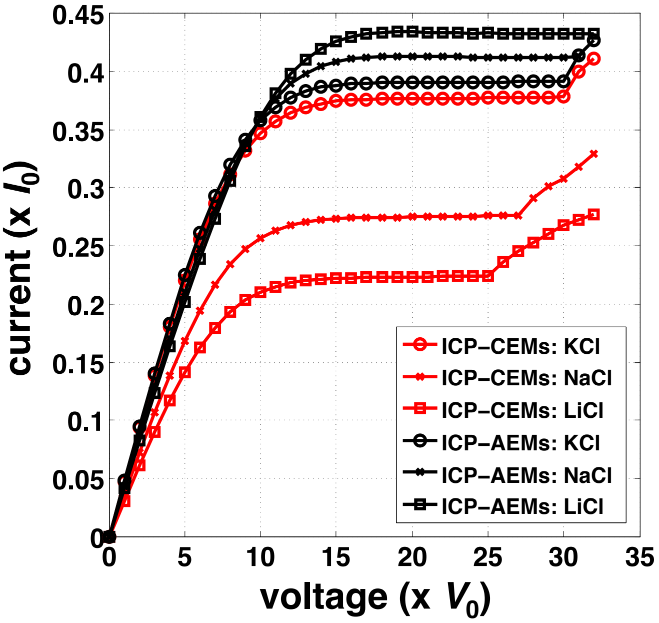

SI Figure 8 | Current-Voltage (I-V) curve of unipolar ICP desalination. a, I-V curves of ICP-CEMs and ICP-AEMs with various salts, 10 mM KCl, NaCl, LiCl. Average flow velocity of simulation model is U_{HP}=800U_0 (U_0=9.89 μm/s). V_0 is thermal voltage, 25.85mV, and I_0 is 330μA.

4. Visualization of ICP Phenomenon in Experiment

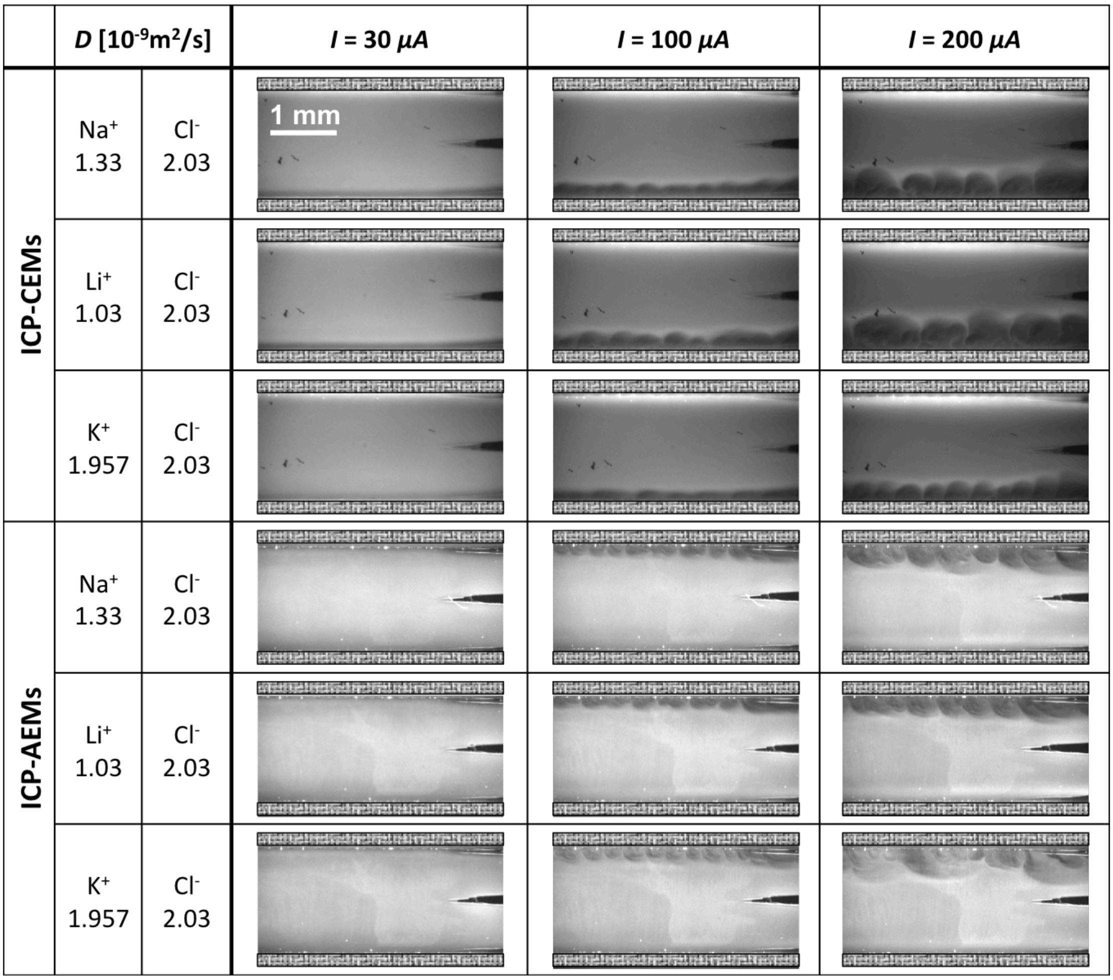

SI Figure 9 | Visualized ion concentration polarization (ICP) in unipolar ICP-experiment. Electric field is applied from up to down, resulting ion depletion on the bottom CEM or on the upper AEM. The meshed boxes indicate CEM in ICP-CEMs or AEM in ICP-AEMs. Ion concentration is visualized by fluorescent dyes: 0.78 μM Alexa Fluor 488 in ICP-CEMs and 5 μM rhodamine 6g (R6G) in ICP-AEMs

5. Experimental Comparative Analysis between ED and ICP Systems

5.1. Experimental setup

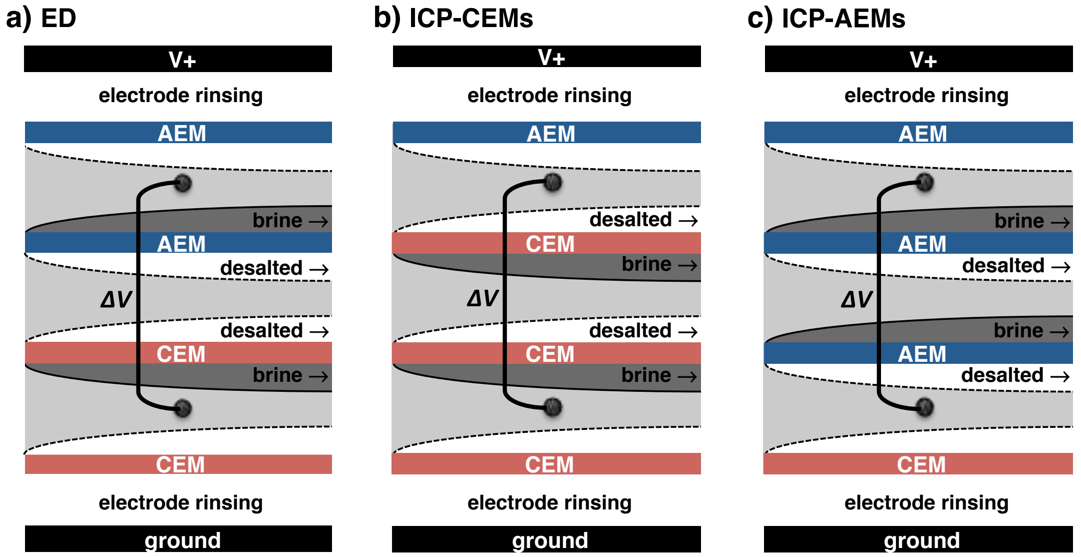

SI Figure 10 | Schematic experimental setup of **a,** ED, **b,** ICP-CEMs, and **c,** ICP-AEMs. White regions indicate desalted flows and dark gray regions indicate brine flows. During constant current is applied by electrodes (black boxes), two probes (black dots) measure the voltage drops in one membrane pair (N=1), ΔV.

To demonstrate and characterize unipolar ICP system and compare it with standard bipolar ED (in Fig.3-5), the device is fabricated by slotting IEMs and electrodes into Polydimethylsiloxane (PDMS), as described in Fig. 2a and Kwak *et.al*[19]. ED and ICP systems can be stacked, so we select one membrane pair (N=1) of the systems, and put them between the AEM-anode and the CEM-cathode (SI Fig. 10). To measure conductance, in ED system, we collect two desalted flows on one AEM and one CEM together in one channel (SI Fig. 10a). In ICP system, the conductance of one desalted flow on the lower CEM (or AEM) is gathered and gauged, but we let the other desalted flow on the upper CEM (or AEM) flow (SI Fig. 10b-c). The recovery ratio is fixed as 50% by holding the same desalted and brine flow rates.

In this experiment, the pumping power W_{pump} can be calculated by multiplying total flow rate of desalted and brine flows Q_{total} (here, 100 μL/min) and the pumping pressure P. From the equation of Hagen-Poiseuille flow[20], the pressure drop in rectangular channel with length L, width w, and depth d is;

$$P = \frac{12\mu Q_{total} L}{wd^3}, \qquad \text{(SI 5.1)}$$

where μ is dynamic viscosity of water. Then, with 50% water recovery, the pumping energy to produce desalted water flow rate Q_{desalt} becomes

$$W_{pump} / Q_{desalt} = \frac{Q_{total} P}{Q_{desalt}} = 0.5P = \frac{6\mu Q_{total} L}{wd^3},\ Q_{total} / Q_{desalt} = 0.5\ . \quad \text{(SI 5.2)}$$

By substituting the parameters in the experiment (*L*: 20mm, *w*:2mm, *d*:0.6mm),

$$W_{pump} / Q_{desalt} = \frac{6 \times 0.001[kg / m \cdot s] \times 100[\mu L / \min] \times 20[mm]}{2[mm] \times (0.6[mm])^3} = 0.103[\mu Wh / L]\ . \quad \text{(SI 5.3)}$$

It is noted that the pumping energy is negligible compared to electric energy consumption (Fig. 5e), because ICP desalination is not a filtration process such as shock ED[21] or reverse osmosis (RO)[22].

5.2. Characteristics of current-voltage curves

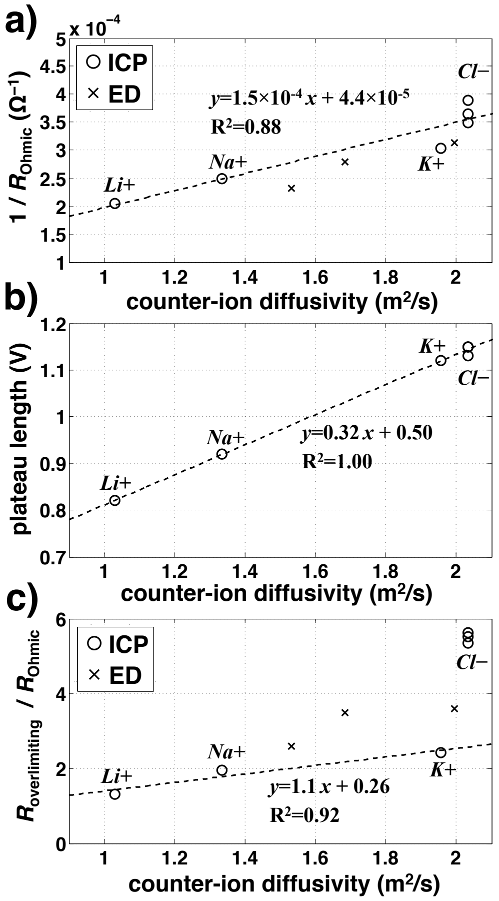

SI Figure 11 | Characteristics of current-voltage (I-V) curves of ICP and ED systems. a, the resistance in Ohmic regime R_{Ohmic}, **b,** the plateau length of limiting reigme, and **c,** the resistance ratio in overlimiting R_{over} and Ohmic regime R_{Ohmic}, according to counter-ion diffusivities (ICP-CEMs: cation diffusivity D_+, ICP-AEMs: anion diffusivity D_-). The averaged diffusivity $(D_+ + D_-)/2$ is used to plot ED cases. Dotted lines indicate the best linear fitting curves. As described by linear ICP model, the limiting current and corresponding slope (*i.e.* $1/R_{\text{Ohmic}}$) is linearly proportional to the diffusivity of the permeable counter-ion (SI Fig. 11**a**). Therefore, ICP-AEMs with Cl- has lower resistance in Ohmic regime than ICP-CEMs. However, this tendency of resistance is flipped in overlimiting regime; slower counter-ion facilitates electroconvection, resulting shorter plateau length (SI Fig. 11**b**) and lower $R_{\text{overlimiting}}$ (SI Fig. 11**c**).

6. Characterization of Desalination Performance

6.1. Basic equations for quantifying desalination performance

To compare two types of unipolar ICP desalination (with CEMs and AEMs) and bipolar ED quantitatively, we control or trace all parameters in the systems, including current or voltage, conductivity, flow rate, device's geometry, *etc.*. We calculate five metrics: **i)** salt removal ratio, **ii)** energy consumption, **iii)** energy per ion removal (EPIR), **iv)** current utilization (CU). Here, we define these metrics, relations between metrics, and meanings clearly, while some of them are described above.

First, salt removal ratio is a parameter to indicate the desalting ability of devices. By measuring the concentration (or conductivity) of sample flows C_0 and that of the desalted flow C_{desalted}, we can figure out how many salt ions are removed from the desalted flow. Salt removal ratio can be given by the ratio between concentration drop and the initial ion concentration:

$$\text{Salt removal ratio} = \frac{C_0 - C_{desalted}}{C_0}. \qquad \text{(SI 6.1)}$$

The concentrations can be converted from the measured conductivity σ with given molar conductivity Λ of electrolytes. Here, we use only dilute monovalent binary electrolytes ($z_+ = z_- = 1$), 10 mM KCl, NaCl, and LiCl solutions. Then, the equation for the conversion is

$$C_i[mol/m^3 = mM] = \frac{\sigma}{\Lambda_{+,i} + \Lambda_{-,i}}[\frac{S/m}{S \cdot m^2/mol}]. \qquad \text{(SI 6.2)}$$

where $\Lambda_{+,i}$ and $\Lambda_{-,i}$ are the molar conductivity of cation and anion *i*. The molar conductivities of Cl^-, K^+, Na^+, Li^+ are 7.63, 7.36, 5.01, and 3.87 [10^{-3} Sm^2mol^{-1}][3], respectively.

To compare different desalination devices, energy consumption has been frequently measured. In electrochemical desalination systems, energy consumption is evaluated by the electrical power consumption (*IV*) divided by the flow rate of desalted water Q_{desalted};

$$\text{Energy consumption} = \frac{IV}{Q_{desalted}}[Wh/L]. \qquad \text{(SI 6.3)}$$

While energy consumption is an important metric determining the economic cost of the desalination technique, it cannot represent the ability of removing salt. We therefore consider energy consumption to remove a single pair of cation and anion, *i.e.* EPIR:

$$\text{Energy per ion removal } = \frac{IV / Q_{desalted}}{zk_B T(C_0 - C_{desalted})}. \qquad \text{(SI 6.4)}$$

EPIR is a parameter representing how efficiently energy is consumed to reject ions. However, it is noted that salt removal ratio or the value of conductivity drop should be checked together, because better EPIR does not necessarily represent better desalting performance (*i.e.* the trade-off of throughput and EPIR discussed in the main text).

Current utilization (CU) describes the ratio of rejected ions in desalted flow and ions transferred at the electrodes. The following equation Eq. SI 6.5 is a modified one to obtain CU from the concentration differences of initial sample flow and desalted flow;

$$\text{current utilization (CU)} = \frac{zFQ_{desalted}(C_0 - C_{\text{desalted}})}{NI}. \qquad \text{(SI 6.5)}$$

Last, required area represents the area to remove a designated salt removal, which directly represent the systems' throughput:

$$\text{required area } = \frac{A}{C_0 - C_{desalted}}[mm^2 / mM], \qquad \text{(SI 8.6)}$$

where A is the working area of IEM here. The most significant cost of an electrochemical desalination system is the membrane cost; therefore lower required area would be economically favorable. However, there is usually a trade-off between required area and power consumption; if one decreases required area to enhance salt removal ratio with a limited size device by applying higher electric potential, power consumption will increase. If one uses a larger system for better salt rejection at a fixed voltage or current, required area becomes higher.

6.2. Desalination metrics in experiment

To calculate these metrics in the experimental setup (Fig.3a and SI Fig. 10), we record voltage responses (SI Fig. 12a-c), conductivity drop of desalted flows under a constant applied current (Ohmic-limiting: 0.1-0.2mA, overlimiting: 0.2-0.5mA) and a

given flow rates (100 μL/min) of various aqueous solutions with 10 mM KCl, NaCl, and LiCl. Based on the given, controlled, and measured parameters, we obtain salt removal ratio (Fig.3b), CU (Fig.3c), power consumption (SI Fig. 12d-f), and EPIR (SI Fig. 12g-i) for all data points. This extensive dataset with three different electrolytes reveals many interesting trends, and elucidates the differences between unipolar ICP and bipolar ED platforms.

First, the voltage-current responses show the similar tendencies, which described in Fig. 5a (SI Fig. 12a-c). The curves in the same electrolyte cross at 0.2-0.3mA. Correspondingly, the power consumptions of three systems also meet at the same current (SI Fig. 12d-f). In the case of ICP-AEMs, in Ohmic regime (<0.2mA) chlorine ions can move faster with higher molar conductivity, resulting in lower electric resistance and lower voltage drop response at a given current (SI Fig. 12a-c), and lower power consumptions (SI Fig. 12d-f) than the other two systems. However, salt removal ratio of ICP-AEMs are worse than the others because it picks worse ion depletion zone (Fig.3b), under the same operating current; ICP-CEMs shows larger salt removal ratio than the others, meaning that ion depletions on CEMs can remove more ions from desalted flow under the same amount of driving current. It is noted that, with slower cation (K^+> Na^+> Li^+), the salt removal ratio increases for ICP-CEMs, and decreases for ICP-AEMs (Fig.3b). In contrast, the salt removal ratio of ED is independent of the cation used with fixed CU=1 (ideal cases). In this scenario, all three systems have similar EPIR values both at a given current (SI Fig. 12g-i) and at a given salt removal ratio (Fig.5d) in Ohmic regime; because EPIR represents the combined efficiency of both power consumption and salt removal.

In overlimiting regime, however, ICP-AEMs show the highest required voltage (SI Fig. 12a-c) and power consumption (SI Fig. 12d-f), even it still has lowest salt removal ratio (Fig.3b). As described in Fig.5, this is due to the weaker electroconvection and non-ideal effects (water splitting, current-induced membrane discharging, and fouling). As a result, EPIR of ICP-AEMs is significantly upshifted from that of ICP-CEMs.

In all three systems, conducting slower ions (Li^+) require more power than faster ions (K^+ and Na^+) (SI Fig. 12d-f). EPIR in overlimiting regime is typically around few hundred k_BT, and it becomes ~O($10k_BT$) in Ohmic regime, which is comparable with state-of-the-art capacitive deionization (CDI) systems[23]. While the operation in Ohmic regime (< 50 μA) shows better energy efficiency (*i.e.* EPIR)(SI Fig. 12g-i), the

required area is significantly higher (SI Fig. 12j-l). This enlightens us about the trade-off in optimization of desalting processes; better EPIR and worse required area (indicating low flow rate or large size of device)(*e.g.* CDI, Ohmic ED, Faradic ICP desalination)[23-25], or higher salt removal ratio and lower required area but worse EPIR (*e.g.* overlimiting ED and overlimting ICP desalination)[19, 21, 26]. The former is ideal for achieving the maximum energy efficiency, but challenging to deal with large amount of salts (high salinity feed water). The latter can handle high salinity feed water (due to high salt removal ratio) and the system size can be minimized, at the cost of higher energy expense per ions removed.

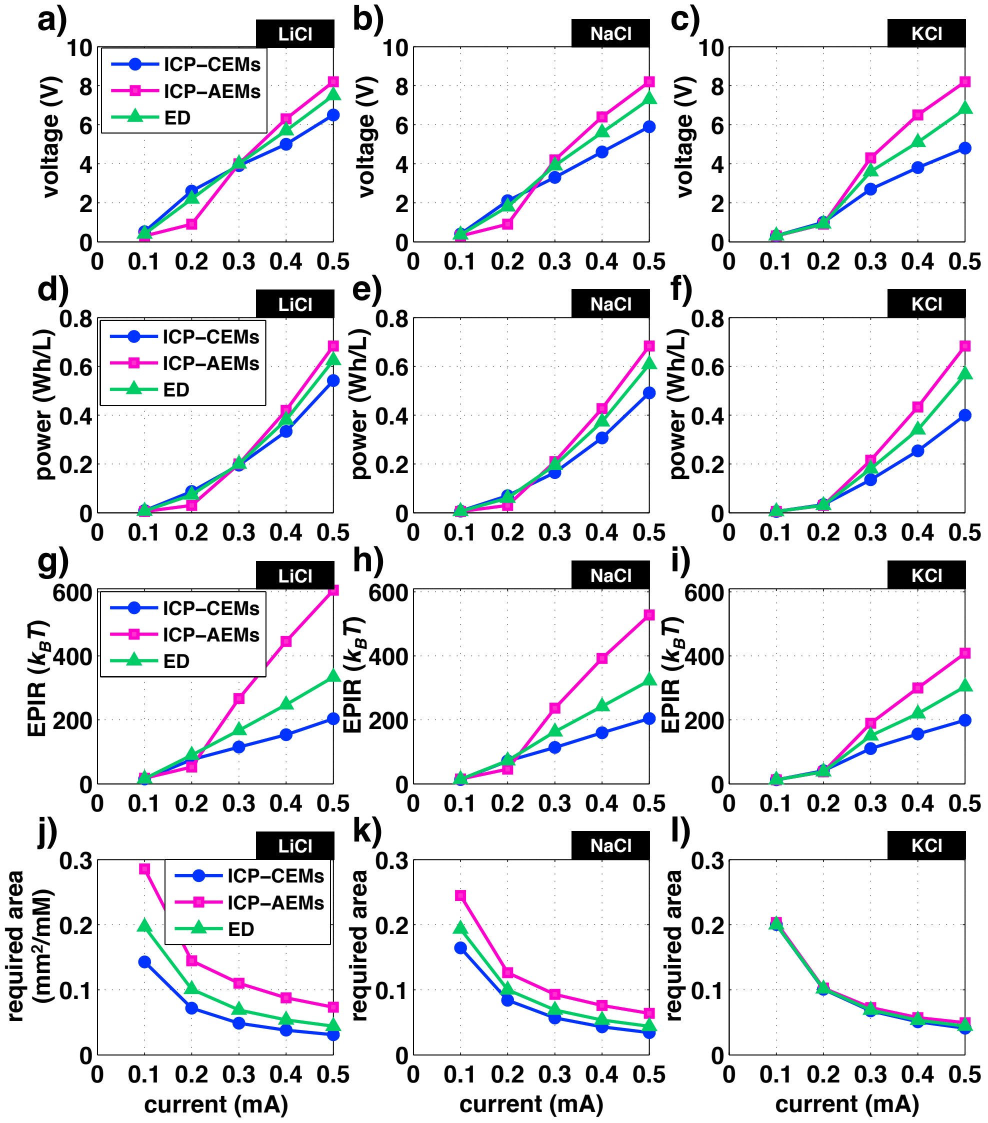

SI Figure 12 | Desalination metrics of ICP and ED systems in various electrolytes. a-c, Voltage responses when the constant current is applied (0.1-0.5mA), **d-f**, power consumption, **g-i,** EPIR, and **j-l,** required area of ICP-CEMs (blue), ICP-AEMs (red) and ED (green), according to the electrolytes, KCl, NaCl, and LiCl.

7. Extended Platforms of Unipolar ICP System for Various Applications

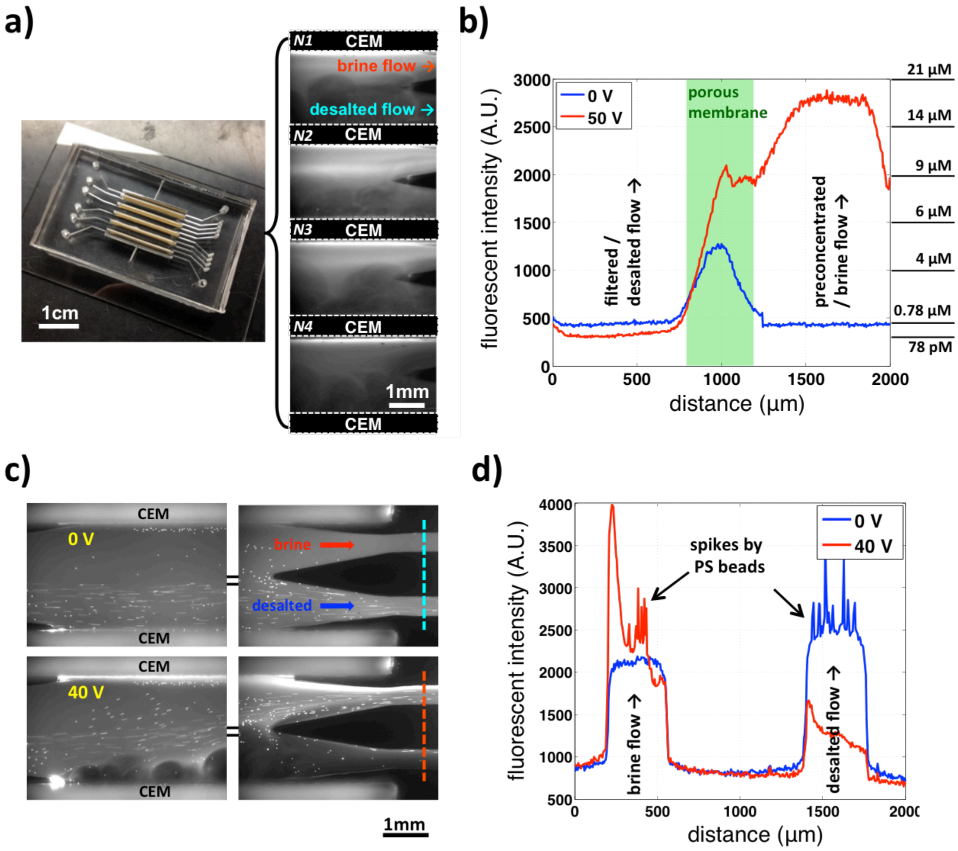

SI Figure 13 | Various modified platforms and tests of unipolar ICP system. a, 4-channel scaled-up system, **b**, fluorescent intensity curve in membrane-added platform (Fig. 6c), and **c-d**, fluorescent image (**c**) and its intensity curve (**d**) in *En Bloc* removal test (Fig. 6d).

4-channel design (*N1-N4*) of ICP-CEMs is demonstrated with 1mm channel depth (2 mm width and 10 mm length)(SI Fig. 13a). Ion depletions on CEMs are well developed in all four channels. This design can be achieved ~1mL/min with rejection of most salts at 100V (~25V per channel); ion depletion zones in each channel are developed up to (or above) the bifurcation points of desalted and brine flows. Sheared electroconvective vortices (EC) seem more chaotic then sallow channel case (0.2 mm height) because of 3-D effect[27].

In SI Fig. 13b, fluorescent intensity curve is measured right before the flow bifurcation for ~95% water recovery / 21-fold preconcentration demonstration (Fig. 6c). The rejection of dyes in desalted flow (0.78 µM→ < 0.78 pM) and the enrichment in brine or preconcentrated flow (0.78 µM→ < 18 µM, *i.e.* ~23-fold preconcentration) are clearly observed.

SI Fig. 13c-d is the demonstration of *En Bloc* removal with negatively charged particles and dyes in ICP platform. It is noted that bio-agents in nature are generally negatively charged (*e.g.* Escherichia coli, Salmonella)[28]. Fluorescent images and its intensity curve after flow bifurcation is shown when voltage is applied or not. The fluorescent particles (only loaded in desalted flow) and dyes are pushed away from the lower CEM; as a result, dark region (low fluorescent intensity) of the desalted flow and bright region (high fluorescent intensity) of the brine flow are observed. The spikes on the fluorescent intensity curves indicate the existence of particles.

Reference

1. J. Newman and K. E. Thomas-Alyea, *Electrochemical Systems*, Wiley-Interscience, New York, 3rd edn., 2004.
2. D. A. Vermaas, D. Kunteng, M. Saakes and K. Nijmeijer, *Water Res*, 2013, **47**, 1289-1298.
3. R. F. Probstein, *Physicochemical Hydrodynamics: An Introduction*, Wiley-Interscience, New York, 2nd edn., 2003.
4. L. Marder, A. M. Bernardes and J. Z. Ferreira, *Sep Purif Technol*, 2004, **37**, 247-255.
5. M. Sadrzadeh and T. Mohammadi, *Desalination*, 2009, **249**, 279-285.
6. Y. J. Kim and J. H. Choi, *Sep Purif Technol*, 2010, **71**, 70-75.
7. A. A. Jalali, F. Mohammadi and S. N. Ashrafizadeh, *Desalination*, 2009, **237**, 126-139.
8. Y. Tanaka, R. Ehara, S. Itoi and T. Goto, *J Membrane Sci*, 2003, **222**, 71-86.
9. R. Kwak, V. S. Pham, K. M. Lim and J. Y. Han, *Phys Rev Lett*, 2013, **110**.
10. V. S. Pham, Z. Li, K. M. Lim, J. K. White and J. Han, *Phys Rev E*, 2012, **86**, 046310.
11. S. V. Patankar and D. B. Spalding, *Int J Heat Mass Tran*, 1972, **15**, 1787-&.
12. J. H. Ferziger and M. Peric, *Computational Methods for Fluid Dynamics*, Springer, 3rd ed. edn., 2001.
13. C. M. Rhie and W. L. Chow, *AIAA Journal*, 1983, **21**, 1525-1532.
14. M. Darwish, I. Sraj and F. Moukalled, *J Comput Phys*, 2009, **228**, 180-201.
15. J. B. S. Balay, K. Buschelman, V. Eijkhout, W. Gropp, D. Kaushik, and L. C. M. M. Knepley, B. Smith, and H. Zhang, *PETSc users manual*, Mathematics and Computer Science Division, Argonne National Laboratory, June 2012.
16. C. Geuzaine and J. F. Remacle, *Int J Numer Meth Eng*, 2009, **79**, 1309-1331.
17. I. Rubinstein and B. Zaltzman, *Phys Rev E*, 2000, **62**, 2238-2251.
18. V. S. Pham, Z. R. Li, K. M. Lim, J. K. White and J. Y. Han, *Phys Rev E*, 2012, **86**.
19. R. Kwak, G. F. Guan, W. K. Peng and J. Y. Han, *Desalination*, 2013, **308**, 138-146.
20. D. J. Beebe, G. A. Mensing and G. M. Walker, *Annu Rev Biomed Eng*, 2002, **4**, 261-286.
21. D. S. Deng, E. V. Dydek, J. H. Han, S. Schlumpberger, A. Mani, B. Zaltzman and M. Z. Bazant, *Langmuir*, 2013, **29**, 16167-16177.
22. M. Elimelech and W. A. Phillip, *Science*, 2011, **333**, 712-717.
23. R. Zhao, P. M. Biesheuvel and A. van der Wal, *Energ Environ Sci*, 2012, **5**, 9520-9527.
24. H. Strathmann, *Desalination*, 2010, **264**, 268-288.
25. K. N. Knust, D. Hlushkou, R. K. Anand, U. Tallarek and R. M. Crooks, *Angew Chem Int Edit*, 2013, **52**, 8107-8110.
26. S. J. Kim, S. H. Ko, K. H. Kang and J. Han, *Nat Nanotechnol*, 2010, **5**, 297-301.
27. E. A. Demekhin, N. V. Nikitin and V. S. Shelistov, *eprint arXiv:1402.2889*, 2014.
28. K. A. Soni, A. K. Balasubramanian, A. Beskok and S. D. Pillai, *Curr Microbiol*, 2008, **56**, 93-97.
